# Supplementary figures and images for: Transferable Coarse-Grained Potential for De Novo Protein Folding and Design
Source: PLoS One. 2014 Dec 1;9(12):e112852. doi: 10.1371/journal.pone.0112852 (PMC4249799; doi:10.1371/journal.pone.0112852)

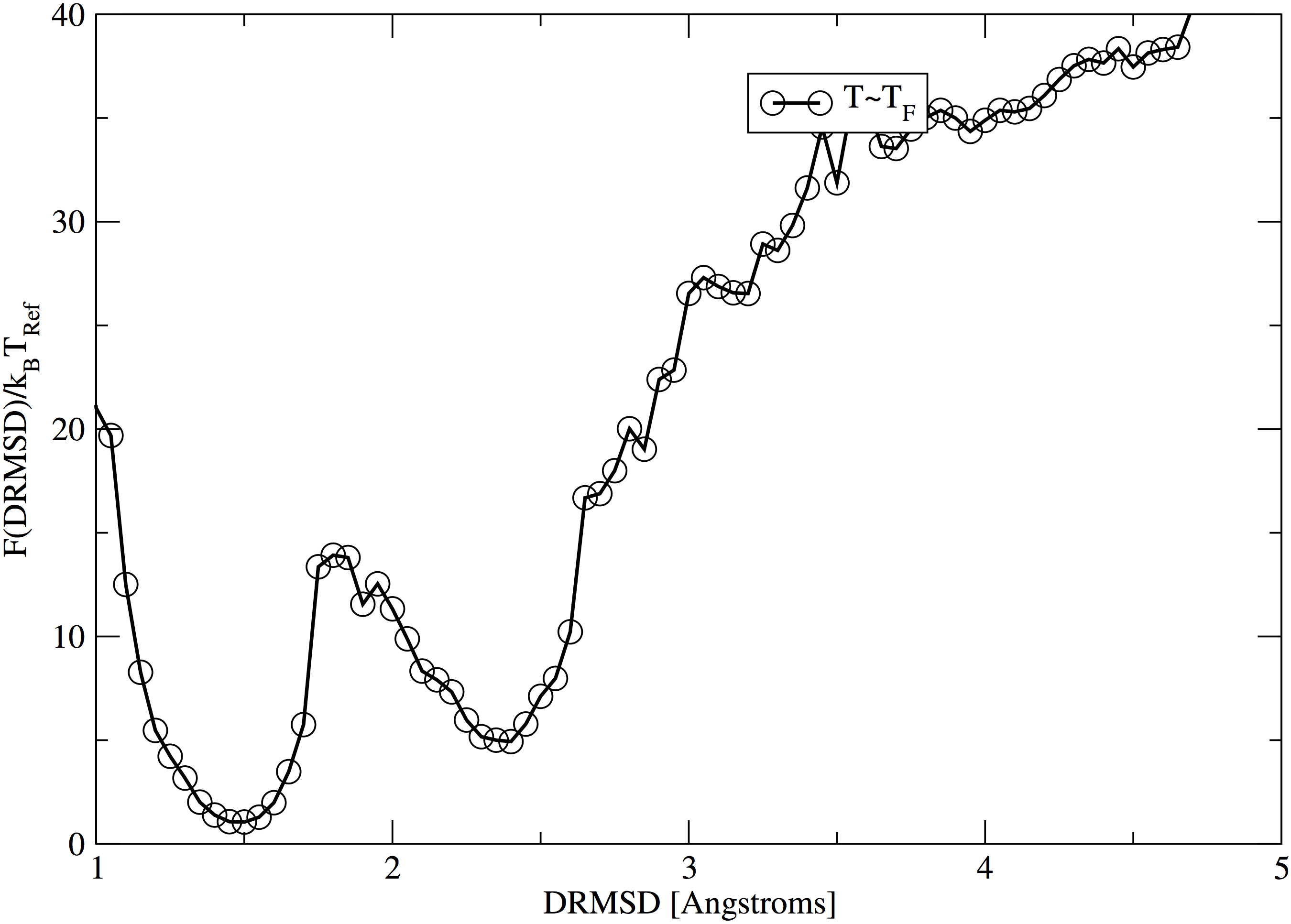

Supplement: Figure S1 — Folding free energy landscape as a function of DRMSD of the designed protein PDB ids 1CTF close to the folding temperature. (TIFF) [file pone.0112852.s001.tiff]

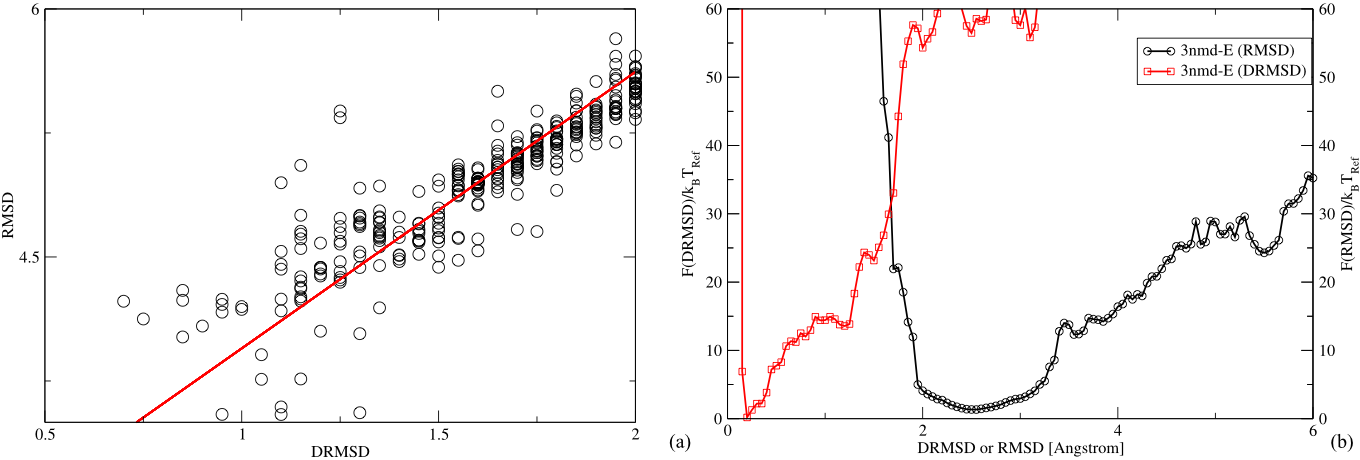

Supplement: Figure S2 — On the left: correlation plot between the DRMSD and the RMSD collective variable. The estimated correlation coefficient from a linear regression fitting (in red) is ≈0.8 which increases to ≈0.98, if we exclude the configurations for values of DRMSD <1.5 Å which is below the model resolution, indicating that the free energy profile should be qualitatively similar if the states are projected over RMSD instead of DRMSD. On the right: Free Energy folding profile of the protein 3NMD-E projected over the collective variables DRMSD and RMSD. The profiles are not identical because the RMSD is more sensitive to local distortions of the protein with respect to the DRMSD. This is also demonstrated by the wider free energy minimum which reflects the thermal fluctuations. However, overall the qualitative shape of the profiles is very similar with between each other in particular since both have a clear global free energy minimum. (TIFF) [file pone.0112852.s002.tiff]

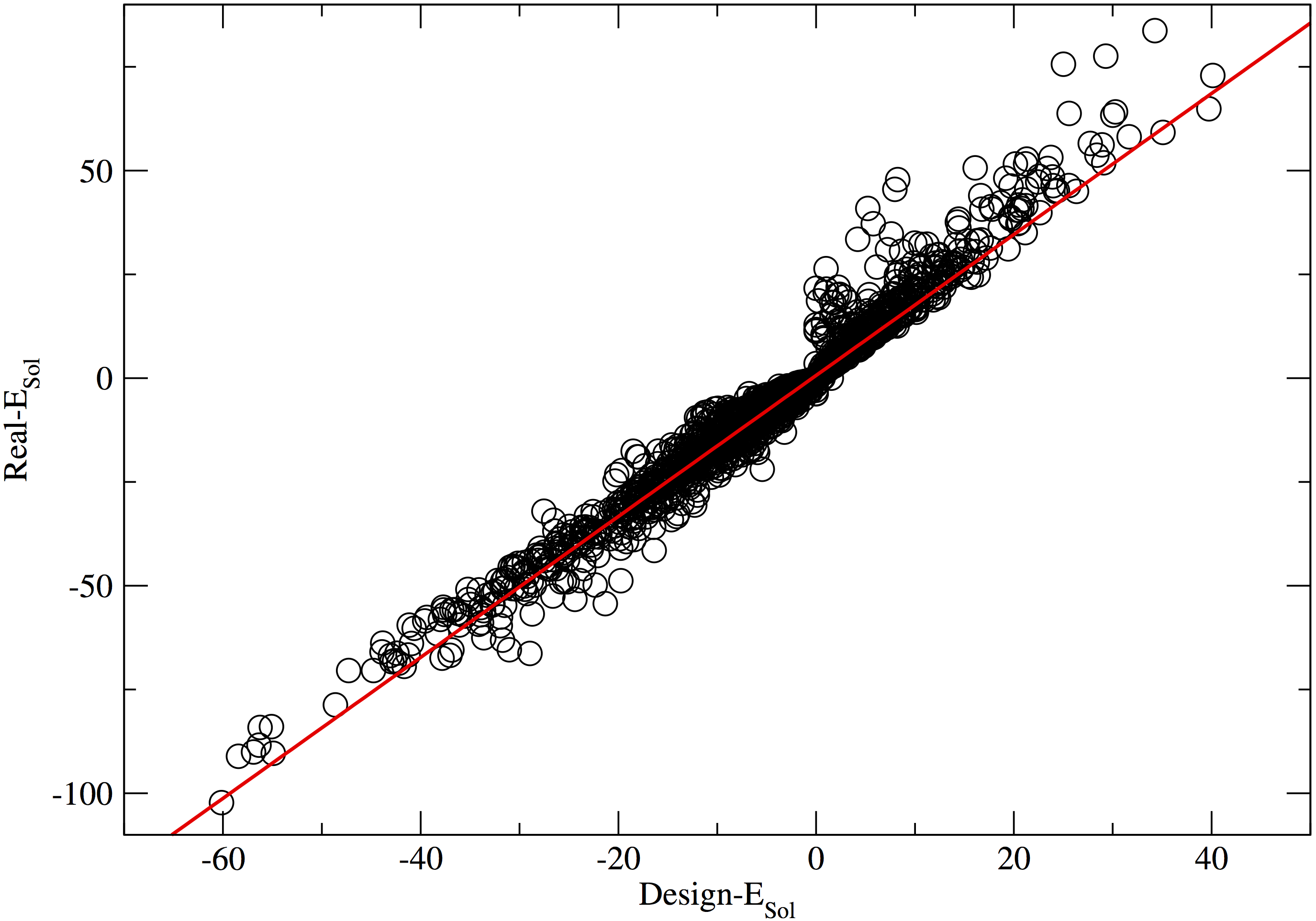

Supplement: Figure S3 — Correaltion between designed and real profiles. The correlation coefficient has been estimated from a linear regression fitting (in red) to be be very high ≈0.98. (TIFF) [file pone.0112852.s003.tiff]

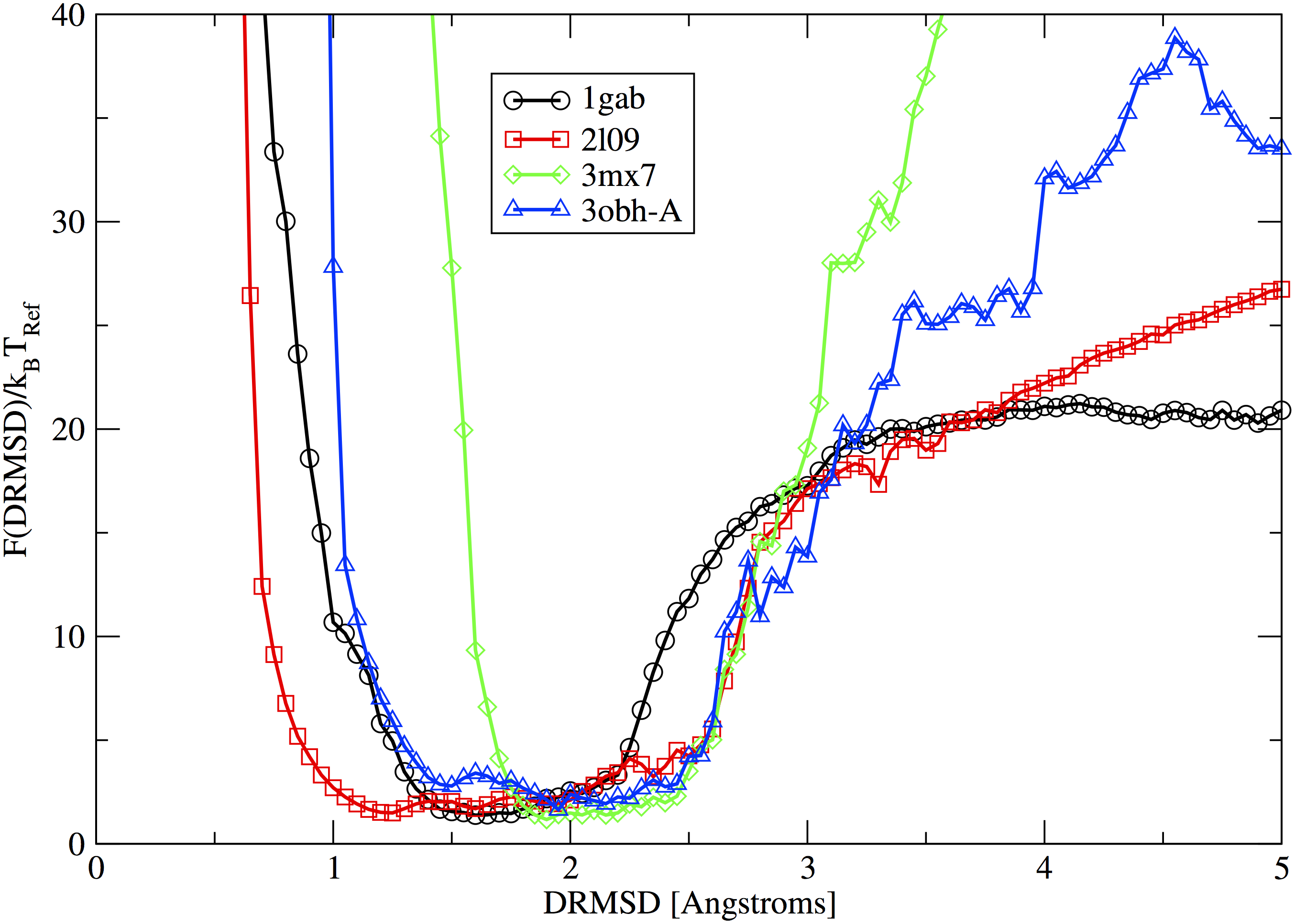

Supplement: Figure S4 — Folding free energy landscape as a function of DRMSD of the four designed proteins (PDB ids 2l09, 3mx7, chain A of 3obh, and 1qyp). All profiles have a global minimum around 1.5 and 2 Å DRMSD with a smooth funnelled shape. Due the approximations present in the model and to thermal fluctuations is shifted with respect to DRMSD = 0 (note that to the value DRMSD = 0 of each profile will correspond a different native structure). Because of the definition of DRMSD, the smaller the value the fewer are the possible structures that can have this value of DRMSD. Ultimately, DRMSD = 0 is possible only for the target structure itself. The funnelled profiles with single minimum implies that both an ensemble of arrested structures and a single alternative fold are less stable compared to the desired configuration. In the bottom right inset we plot the folding free energy landscape for 3mx7 as a function of both the DRMSD and the number of hydrogen bonds , to give a visual example of the funnel nature of the folding landscapes. On the left we compare the experimentally determined structures (in yellow) with a typical folded conformation selected as the sampled configurations with the lowest energy at the free energy minimum (in red). The RMSD value is indicated in the middle. (TIFF) [file pone.0112852.s004.tiff]
