# Supplementary material for: Transferable Coarse-Grained Potential for De Novo Protein Folding and Design
Source: PLoS One. 2014 Dec 1;9(12):e112852. doi: 10.1371/journal.pone.0112852 (PMC4249799; doi:10.1371/journal.pone.0112852)
Supplement: Table S1 — Optimized values of the residue-solvent and residue-residue interaction parameters. The uncertainty on the values is ≈±0.01. (PDF) [file pone.0112852.s005.pdf]

TABLE S1: Optimized values of the residue-solvent ϵ_{Sol} and residue-residue $\epsilon(S_k)(S_l)$ interaction parameters. The uncertainty on the values is $\approx \pm 0.01$

Restype	ϵ_{Sol}	$\epsilon(S_k)(S_l)$																			
ALA	1.80	0.10																			
CYS	2.50	-0.10	0.39																		
ASP	-3.50	-0.70	-0.09	-0.23																	
GLU	-3.50	-0.23	-0.44	-0.17	-0.32																
PHE	2.80	-0.22	-0.92	0.41	0.32	-0.33															
GLY	-0.40	-0.45	-0.18	0.51	-0.00	-0.51	-0.37														
HIS	-3.20	0.17	0.89	-0.89	0.87	-0.82	-0.05	0.63													
ILE	4.50	0.29	0.06	-0.02	-0.51	-0.59	0.08	-0.77	-0.38												
LYS	-3.90	-0.02	0.63	0.13	-0.47	-0.85	-0.99	-0.52	0.23	0.19											
LEU	3.80	-0.50	-0.11	-0.27	-0.55	-0.22	-0.33	-0.36	-0.40	0.60	0.24										
MET	1.90	0.46	0.67	0.59	-0.92	-0.29	-0.48	-0.26	0.02	0.69	0.01	0.89									
ASN	-3.50	-0.76	1.04	-0.15	-0.93	0.14	0.20	-0.34	-0.37	-0.90	0.08	0.53	0.33								
PRO	-1.60	-0.72	0.49	0.03	-0.46	-0.64	0.82	-0.19	0.05	0.80	-0.70	-0.41	-0.48	-0.57							
GLN	-3.50	-0.83	0.09	-0.29	-0.64	-0.35	-0.69	0.17	0.30	-0.87	-0.80	0.68	-0.66	0.74	0.34						
ARG	-4.50	0.36	0.40	-0.73	0.56	-0.80	-0.60	-0.94	-0.38	-0.12	-0.88	-0.49	-0.85	-0.79	-0.20	0.12					
SER	-0.80	-0.62	-0.76	-0.39	-0.14	-0.18	0.03	-0.78	-0.20	-0.85	-0.54	0.65	-0.01	-0.39	0.16	0.35	0.27				
THR	-0.70	-0.12	-0.99	-0.24	-0.44	-0.52	-0.63	-0.74	0.57	-0.07	-0.49	-0.07	0.91	0.53	-0.78	0.26	0.15	0.54			
VAL	4.20	0.42	-0.63	-0.47	0.09	0.37	-0.97	0.11	-0.06	-0.56	-0.35	-0.16	0.25	-0.81	-0.14	-0.53	-0.96	-0.58	-0.03		
TRP	-0.90	-0.60	-0.02	0.23	-0.29	0.48	0.10	0.50	-0.42	-0.47	-0.31	-0.68	0.51	-0.09	1.07	-0.55	0.93	-0.22	-0.72	0.28	
TYR	-1.30	-0.57	0.85	0.06	-0.74	-0.10	-0.99	-0.26	-0.66	-0.98	0.87	-0.60	-0.32	0.96	0.97	-0.96	0.43	-0.89	0.30	0.21	0.33
		ALA	CYS	ASP	GLU	PHE	GLY	HIS	ILE	LYS	LEU	MET	ASN	PRO	GLN	ARG	SER	THR	VAL	TRP	TYR
