# Supplementary material for: Transferable Coarse-Grained Potential for De Novo Protein Folding and Design
Source: PLoS One. 2014 Dec 1;9(12):e112852. doi: 10.1371/journal.pone.0112852 (PMC4249799; doi:10.1371/journal.pone.0112852)
Supplement: Table S2 — List of PDB id's used as training set for the maximum entropy parameters optimization. (PDF) [file pone.0112852.s006.pdf]

TABLE S2: List of PDB id's used as training set for the maximum entropy parameters optimization

1WVH	1MSI	1LU4	1IFC	3EAZ
3B79	3EUR	1TUA	1ZCE	3JVE
1ZHV	2VQ4	2BVV	1T3Y	1TP6
3Q6L	3CX2	3F2Z	3HP4	2GRC
2E3H	2GS5	1N7E	2ESO	2WJ5
2VY8	2GZQ	1AMM	1HK0	3KZD
1O8X	1ZLM	3CO1	1YZM	2PPO
2QVK	2X25	3NZL	3HNX	1I2T
2LIS	1FL0	3NGP	1L3K	3ICH
2X5Y	1JL1	1F21	3I35	1BKR
2QVG	1YU7	1HZT	2F4K	3MSI
1LN4	2NRR	1ZEQ	1TZV	2YV0
2RN2	1EW4	1P7S	2ON8	1OGW
2GI9	3EY6	2RB8	1NG6	1X3O
1IGD	2O37	2NR7	2FG1	2FB6
3EYE	3IV4	2WWE	1QAU	3DVW
1ULR	1YU5	2V4X	3A2Z	1NA5
2JLI	3Q6L	3BZT	3DFG	3KB5
1G9O	1Z21	3OBS	1LMI	3BZP
2NT3	1P5F	2FQ3	3BZS	3S4M
3GBW	1UKF	2VWR	2OZF	2IWR
2IWN	2GZV	3LAX	3A7L	2B02
3I2V	2VC8	1Y0M	2PTH	2JIC
3I7M	2VH7	2END	1HKA	3K0N
3K0M	2WLW	2F1S	3CTG	1XAW
