# Supplementary material for: Transferable Coarse-Grained Potential for De Novo Protein Folding and Design
Source: PLoS One. 2014 Dec 1;9(12):e112852. doi: 10.1371/journal.pone.0112852 (PMC4249799; doi:10.1371/journal.pone.0112852)
Supplement: Table S3 — Comparison of the average composition of the designed sequences and the natural sequences used in the parameter optimization. It is important that since we do not model Cys-Cys bond and the Proline rigid bond we have excluded them from the design alphabet. This is why the frequency associated to those amino acids is zero in the designed sequences. We are currently working on implementing such special cases in the Caterpillar model. We have highlighted in bold the amino acids types with the largest discrepancies namely: Histidine, Methionine, Tryptophan, Tyrosine. Such amino acids are know to be the one with the lowest appearance frequency in nature. Since we did not impose any restriction on the design procedure over the relative abundance of amino acids in nature it is not surprising to find the largest discrepancies in the composition for such amino acids. (PDF) [file pone.0112852.s007.pdf]

TABLE S3: Comparison of the average composition of the designed sequences and the natural sequences used in the parameter optimization. It is important that since we do not model Cys-Cys bond and the Proline rigid bond we have excluded them from the design alphabet. This is why the frequency associated to those amino acids is zero in the designed sequences. We are currently working on implementing such special cases in the Caterpillar model. We have highlighted in bold the amino acids types with the largest discrepancies namely: Histidine, Methionine, Tryptophan, Tyrosine. Such amino acids are know to be the one with the lowest appearance frequency in nature. Since we did not impose any restriction on the design procedure over the relative abundance of amino acids in nature it is not surprising to find the largest discrepancies in the composition for such amino acids.

Residue	Natural Freq	Designed Freq.
A	7.23%	5.13%
C	1.08%	0.00%
D	5.92%	4.99%
E	7.00%	5.61%
F	4.12%	5.86%
G	7.55%	7.74%
**H**	2.28%	6.00%
I	5.79%	4.57%
K	6.75%	7.21%
L	8.94%	4.58%
**M**	1.72%	4.41%
N	4.27%	5.39%
P	4.28%	0.00%
Q	3.98%	5.62%
R	5.13%	6.73%
S	5.90%	4.85%
T	5.79%	5.25%
V	7.39%	5.14%
**W**	1.51%	4.17%
**Y**	3.35%	6.77%
