# Supplementary material for: Transferable Coarse-Grained Potential for De Novo Protein Folding and Design
Source: PLoS One. 2014 Dec 1;9(12):e112852. doi: 10.1371/journal.pone.0112852 (PMC4249799; doi:10.1371/journal.pone.0112852)
Supplement: Table S4 — Designed sequences. (PDF) [file pone.0112852.s008.pdf]

TABLE S4: Designed sequences.

	WDDMIIRRRRFVVYYLWGSMTAEVEAEKGTNGFYYHHHDFGTKKKAQQQSNNL
	WDDMIIRRRRFVVYYLWGSMTAEVEAEKGTNGFYYHHHIFGTKKKAQQQSNNL
	WDDMIIRRRRIVVYYLWGSMTAEVEAEKGTNGFYYHHHFFGTKKKAQQQSNNL
**1gab-A**	WNNEVVQQQGGLLKRAARSSNDDDILHHHVFFTTKKKTEGGFYYYYRRIMMIW
	WSNEVAQQQGGLLKRAARSNNDDDILHHHVFFTTKKKTEGGFYYYYRRIMMIW
	WSNEVVQQQGGLLKRAAISNNDDDILHHHVFFTTKKKTEGGFYYYYRRRMMIW
	WSNEVVQQQGGLLKRAARSDNDDNILHHHVFFTTKKKTEGGFYYYYRRIMMIW
	AAAHHQNNNNFKKKKKKGGFQFQGGGGYYYYIEEEEIMWMFIYRHRRRRDDDDLLHVVLMWWTTTVVQSSSS
	AAAVVQYGGGGGGRRVVMDWWWDIRRRYYYYNMMKEEEENNNYKAHKKKTSSQQQFFFFSDIIHHHHLLLTT
	AAAVVQYGGGGVGRRVVMDWWWDIRRRYYYYNMMKEEEENNNYKAKKKKTSSQQQFFFFSDIIHHHHLLLTT
**1leb-A**	ATAVVQGGGGGGVRRRVRDWWWDDIRIYYNYNMMKAEEEYNNYYAKKKKTTSQQQFFFFSSMIHHHHLLLLT
	ATAVVQYGGAGGGRRVMFFWWWIIRRRYYYYNMMKEEEENNNYKAKKKKTSSQQQVFFDSDDIHHHHLLLTT
	ATAVVQYGGGGGGRRVMFFWWWIIRRRWYYYNMMKEEEENNNYKAKKKKTSSQQQVFFDSDDIHHHHLLLTT
	ATAVVQYGGGGGGRRVMFFWWWIIRRRYYYTNMMKEEEENNNYKAKKKKTSSQQQVFFDSDDIHHHHLLLLT
	WGWVYVVAKKKKKGNFFSSSFIIIHHHHLLLDDDDRRRRRFYYYYGGWTTTMMMEEEEEGNNNQQQQAAAT
	WGWVYVVAKKKKKWNFFSSSFIIIHHHHLLLDDDDRRRRRFYYYYGGGTTTMMMEEEEEGNNNQQQQAAAT
	WTWVYVVAKKKKKGNFFSSSFIIIHHHHLLLDDDDRRRRRFYYYYGGGWTTMMMEEEEEGNNNQQQQAAAT
**1pou-A**	WWSVYVVAKKKKKGNFFSSWFIIIHHHHLLLDDDDRRRRRFYYYYGGGTTTMMMEEEEEGNNNQQQQAAAT
	WWSVYVVAKKKKKGNFFSWSFIIIHHHHLLLDDDDRRRRRFYYYYGGGTTTMMMEEEEEGNNNQQQQAAAT
	WWWQYVVAKKKKKGNFFSSSFIIIHHHHLLLDDDDRRRRRFYYYYGGGTTTMMMEEEEEGNNNQQVQAAAT
	WWWSYVVAKKKKKGNFFSSVFIIIHHHHLLLDDDDRRRRRFYYYYGGGTTTMMMEEEEEGNNNQQQQAAAT
	ADLWWWEVMMMTTTAKVGNNNQQQYYYYFFIISSSDDLLHHHFGGGEEAKKKRRRIR
	AMDWWETKMMVYYYVVKKKAANNTQNGGYTRILDISSSIHHHRRGGGEEQYQYFFFLL
	AWWSWVVYVMMMTTTQQQQNNNLLEGGYYFIISSDDDLHHHFFGGEEAAKKKKRRRI
**1qyp-A**	DAWSWVVYVMMMTTTYQQQNNNALEGGYYFIISSDDLLHHHFFGGEEAAKKKKRRRI
	DDWWAVVYWMMMTTTYQQQNNNVLEGGYYFIISSSDLLHHHFFGGEEAAKKKKRRRI
	DGWSMWVYVMMTTTLYQQQNNNVAEGGYYFIISDSDLLHHHFFGGEEAAKKKKRRRI
	DGWWVVEGMMMTTTAKANVNNSQQQYYYYFFISSDDLLLHHHFGGGEEAKKKRRRII
	WEENNNQQYVGGGTRRRIIIFFGGGMMMYYYYFFHHHHHDDDISRRLLLFLQVVVTKKAAAEEQWWNTAKKKTSSS
	WEENNNQQYVGGGTRRRIIIFFGGGMMMYYYYFFHHHHHDDDISRRLLLFLVVQVTKKAAAEEQWWNTAKKKTSSS
	WEENNNQQYGGGTTRRRIIIFFGGGMMMYYYYFFHHHHHDDDISRRLLLFLQVVVKVKAAAEEQWWNTAKKKTSSS
**1sro-A**	WEENNNQQYGGGTTRRRIIIFFGGGMMMYYYYFFHHHHHDDDISRRLLLFLQVVVVKKAAAEEQWWNTAKKKTSSS
	WEENNNQQYGGGTTRRRIIIFFGGGMMMYYYYFFHHHHHDDDISRRLLLFLVVQVVAKKAAEEQWWNTAKKKTSSS
	WEENNNQQYGGGTTRRRIIIFFGGGMMMYYYYFFHHHHHDDDRSIRLLLFLQVVVVKKAAAEEQWWNTAKKKTSSS
	WEENNNQQYVGGGTRRRIIIFFGGGMMMYYYYFFHHHHHDDDIRSRLLLFLQVVVTKKAAAEEQWWNTAKKKTSSS

	DRIMFFFYYYYKKKKKTGFGGGGGRTTMMVWWSWVLLVVAATSSQQQQAEEEENNNNIIRRHHHHLRDDD
	EITTFFYYYYYKKKKKFGFGGRRRRRRHHHDDDSDLLHVVVTGSSGQAAAQQQNNNNEEIEEIMMMLWWWW
	FITTIFYYYYYKKKKKFGFGGRRRRRRHHHDDDSDLLHVVVTGSSGQAAAQQQNNNNEEEEEIMMMLWWWW
**1utg-A**	HRIMFFFYYYYKKKKKTTFGGGGGRTTMMVWWSWVLLVVAAGSSQQQQAEEEENNNNIIRRHRHHLDDDD
	HRRMFFFYYYYKKKKKTTFGGGGGRTTMMVWWSWVLLVVAAGSSQQQQAEEEENNNNIIIRHRHHLDDDD
	HRTMFFFYYYYKKKKKAGFGGGGGRRTMMVWWSWVLLVVATTSSQQQQAEEEENNNNIIIRHHHHLDDDD
	HRTMFFFYYYYKKKKKAGFGGGGGRRTMMVWWSWVLLVVATTSSQQQQAEEEENNNNIIIRHRHHLDDDD
	EQQQQGAEKKKKNNNNFARRARRIIDDDIHHHHYYFYYFFGGGTTTLLLSWEVVMMSWW
	IAHHHYYFYYFFTRTTGRGGFQQVVVLLVQSSSGGKKKKARREEINNNLDDMAAEWDWM
	IDHHHYYFYYFFTRTTGRGGFQQVVALLVQSSSGGKKKKKRREEINNNLIEWAAMMDWW
**1uxd-A**	IDHHHYYFYYFFTRTTGRGGFQQVVALVLQSSSGGKKKKKRREEINNNLIEWAAMMDWW
	IIAHHYYFYYFFTRTTGRGGFQQVVALVLQSSSGGKKKKKRREEINNNLDEWHAMMDWW
	IIEHHYYFYYFFTRTTGRGGFQQVVALLVQSSSGGKKKKKRREHINNNLDEWAAMMDWW
	IIEHHYYFYYFFTRTTGRGGFQQVVALVLQSSSGGKKKKKRREHINNNLDEWAAMMDWW
	WWWDMMMYYYYYQQNNAAAKKKKTTIHHHDDIISRRRRGGGGSSQVVVFFFLLLNGEEET
	WWWDMMMYYYYYQQNNAAAKKKKTTIHHHIDDISRRRRGGGGSSQVVVFFFLLLNGEEET
	WWWIIMMMYYYYQQNNNAAKKKKGTRRRRSDDDHHHFGGGGTSSQVVVFFLLLIAREEET
**1vif-A**	WWWIIMMMYYYYQQNNNAAKKKKTRRRRTSDDDHHHFGGGGGSSQVVVFFLLLIAREEET
	WWWIIMMMYYYYQQNNNAAKKKKTRTRRRSDDDHHHFGGGGGSSQVVVFFLLLIAREEET
	WWWIIMMMYYYYQQNNNAAKKKKTTRRRRDDSDHHHFGGGGGSSQVVVFFLLLIAREEET
	WWWIIMMMYYYYQQNNNAAKKKKTTRRRRSDDDFHHHGGGGGSSQVVVFFLLLIAREEET
	AKKKTKSSVVVQQQNNAEEGGYFRRIIDDLLHHHFGGGEAMMMLHWWDIRRFYYYNTTKK
	AKKKTSSSFVVQQQNNAEEGGYFVIIDDDLLHHHFGGGEAMMMWWWLIRRRRYYYNTKKK
	AKKKTSSSVVVQQQNNAEEGGYFRRIIDDLLHHHFGGGEAMMMNHWLDIRRFYYYNTTKK
**2cdx-A**	AKKKTSSSVVVQQQNNAEEGGYFRRIIDDLLHHHFGGGEAMMMWHWLDIRRFYYYNTTKK
	AKKKTSSSVVVYQQNNAEEGGYFRRIIDDLLHHHFGGGEAMMMWHWLDIRRFYYYNTTKK
	AKKKTSSVVVVQQQNNAEEGGYFRRIIDDLLHHHFGGGEAMMMWHWLDIRRFYYYNTTKK
	ETKKFSSSNVVQQQQAEETGGYFRRIIDDLLHHHHFGGGTMMMLWWNDIRRFYYYVAKKK
	AAAEAEGYYYFHHHMIIRRRMMMTNNNNNLAKKKKTGGHHHLIRRRKKTTGGGWWWVVVVVQQQQQLLEEEGYYFFFIDDSSSSDDF
	AAAEEAGYYFFHHHMIIRRRMMMTNNNNNLEKKKKTGGHHHLIRRRKKTTGGGWWWVVVVVQQQQQLLAEEGYYYFFIDDSSSSDDF
	AAAEEAGYYYFHHHMIIRRRMMMTNNNNNLEKKKKTGGHHHLIRRRKKTTGGGWWWVVVVVQQQQQLLAEEGYYFFFFDDSSSSDDI
**2kyw-A**	AAAEEEFYYYFHHHMIIRRRMMMTNNNNNLAKKKKTGGHHHLIRRRKKTTGGGWWWYVVVVQQQQQLLKEEGYYFGFIDDSSSSDDF
	AAAEEEFYYYHHHIMIIRRRMMMTNNNNNLAKKKKTGGHHHLIRRRKKTTGGGWWWYVVVVQQQQQLLKEEGYYFFFFDDSSSSDDG
	AAAEEEFYYYHHHIMIIRRRMMMTNNNNNLAKKKKTGGHHHLIRRRKKTTGGGWWWYVVVVQQQQQLLKEEGYYGFFFDDSSSSDDF
	AAAEEEGFYYFHHHMIIRRRMMMTNNNNNLAKKKKTGGHHHLIRRRKKTTGGGWWWYVVVVQQQQQLLKEEGYYFFYIDDSSSSDDF

	DNSSQQVLFLLTRRRRIDDNFNHHHAKKKKTKSQQVVAAGGGGGFFYYYYTIIMEEEWWWMM
	DNSSQQVLFLLTRRRRIDDNHNHHHAKKKKTKSQQVVAAGGGGGFFYYYYTIIMEEEWWWMM
	DNSSQQQLFLLTRRRRIDDNFNHHHAKKKKTKSQVVVAAGGGGGFFYYYYTIIEEEWWWMMM
**2l09-A**	DNSSQQVLFLLTRRRRIDDNFNHHHAKKKKTKSSQVAAVGGGGGFFYYYYTIIMEEEWWWMM
	DNSSQQVLFLLTRRRRIDDNFNHHHAKKKKTKSWQVVAAGGGGGFFYYYYTIIMEEEWWQMM
	DNSSQQVLFLLTRRRRIDDNHNFHHAKKKKTKSQQVVAAGGGGGFFYYYYTIIEEEWWWMMM
	NDSSQQVLFLLTRRRRIDDNHNHHHAKKKKTKSQQVVAAGGGGGFFYYYYTIIMMEEWWWEM
	AMDWWSVVVDSWSSAMAAEEGGGRRRDDLLHLVYQQNNNNQQQKKKKKTTFFFFFIIHHHHYYYYMMTTEEGGGRRII
	AMDWWSVVVDSWSSAMAAEEGGTRRRDDLLHLVYQQNNNNQQQKKKKKTGFFFFFIIHHHHYYYYMMTTEEGGGRRII
	AMMSWWDVVASWSDDDIIRRRGGGEKAMMTYYYYYHHHHILIFFFFFGTKKKAKQNNNNQQQQVVLLSLRRTTGGEEE
**2ptl-A**	AMSWWDVVASSWDLAAAEEEGGGRRRDDHLVLYYQNNNNQQQQKKKKKTTFFFFFLIHWHHYYYVMMTTEEGGGRRII
	AMTSWWDVVASSSDDDIIRRRGGGLEAMMNYYYYYHHHHILFFFFFGGTKKKAKQNNNNQQQQVVLLWHRRTTGKEEE
	DMSSWDVVVSSWDDAAAAEEGGGRRRIWLLMLYYQNNNNQQQQKKKKKTTFFFFGLIHHHHYYYVMMTTEEGGGRRII
	DMSSWVVVASSWDDAAAEEEGGGRRRHNWLLLVLQQNNNNQQQKKKKKTTFFFHHHHIIFFYYYYMMTTEEGGGRRII
	IIRRRTTTMTLNNWWEEEYYYFHDLDLHHHHYYYMMMIIRRRIDDDSSSSFFNNNEEAAAAAKKKKKKVVWWSVVVGGGQQQQQFFGGGL
	IMRRRTTTTTLLNWWEEEYYYFFDDLHHHHHYYYMMMIIRRRIDDDSSSSFFNNNEEAAAAAKKKKKKVVWWSVVVGGGQQQNQFQGGGL
	IMRRRTTTTTLNLWWEEEYYYFFDDLHHHHHYYYMMMIIRRIRDDDSSSSFFNNNEEAAAAAKKKKKKVVWWSVVVGGGQQQNQFQGGGL
**3mx7-A**	IMRRRTTTTTLNLWWEEEYYYFFDDLHHHHHYYYMMMIIRRRIDDDSSSSFFNNNEEAAAAAKKKKKKVVWWSVVVGGGQQQNQFQGGGL
	IMRRRTTTTTLNNWWEEEYYYFHDLDLHHHHYYYMMMIIRRRIDDDSSSSFFNNNEEAAAAAKKKKKKVVWWSVVVGGGQQQQQFFGGGL
	ITRRRTTTMTLNNWWEEEYYYFHDDLLHHHHYYYMMMIIRRRIDDDSSSSFFNNNEEAAAAAKKKKKKVVWWSVVVGGGQQQQQFFGGGL
	ITRRRTTTMTLNNWWEEEYYYFHDLDLHHHHYYYMMMIIRRIRDDDSSSSFFNNNEEAAAAAKKKKKKVVWWSVVVGGGQQQQQFFGGGL
	AAAENNQQQEEENTTKMMYYYGGGGTKKVVVQSSSDDLLHHHRRRIIFFWW
	AAAFNNQQQEEEETTTMMYYYGGGGKKKVVVNSSSDDDLHHHRRRIILFWW
	AAAFNNQQQEEEETTTMMYYYGGGGKKKVVVSSSDDDLLHHHRRRIIFFWW
**3nmd-E**	AAAFNNQQQEEEETTTMMYYYGGGGKKKVVVSSSDDDLLHHHRRRIIFNWW
	AAAFNNQQQEEEETTTMMYYYGGGGKKKVVVSSSDDDLLHHHRRRIINFWW
	AAAFNNYQQEEEETTTMMYYYWGGGKKKVVVQSSSDDLLHHHRRRIIDFNW
	AAAINNQQQEEEETTKMMYYYGGGGTKKVVVQSSSDDLLHHHRRRINFFWW
	TTTTKKKKMSSEEGGGGGWWMVVVVQQQQDLLLHHHHMSIIRRRRAAAAWNNNNDDIDREEYYYYFFFF
	TTTTKKKKMSSEEGGGGGWWMVVVVQQQQDLLLHHHHMSIRRRRRAAAAWNNNNDDIDIEEYYYYFFFF
	TTTTKKKKMSSEEGGGGGWWWVVVVVQQQQDLLLHHHHMMIIRRRRAAAASNNNNDDIDREEYYYYFFFF
**3nrl-A**	TTTTKKKKMSSEEGGGGGWWWVVVVVQQQQDLLLHHHHMMIRRRRRAAAASNNNNDDIDIEEYYYYFFFF
	YYYYFFFFIIIHHHHGGGMMVMTRRRRRDDNNNAGGNWVVEEQQQLDDSWSLLAAAEEQWSTTTKKKKK
	YYYYFFFFIIIHHHHGGGMMVMTRRRRRDDNNNEGGNWTVEEQQQLDDSWSLLAAAAEQWSTTVKKKKK
	YYYYFFFFIIIHHHHGGGMMVMTRRRRRDDNNNEGGNWVEEVQQQLDDSWSLLAAAAEQWSTTTKKKKK

	AAEEQQQETTTTGGGGVLVVLSSDSDDDHHHHHISFFFFIIRRRRRVLWWWMMMYYYYYGGAKKKKKQNNNNE
	AAEEQQQETTTTGGGGVVVVLSSDSDDDHHHHHIIFFFFSIRRRRRLLWWWMMMYYYYYGGAKKKKKQNNNNE
	AAEEQQQETTTTGGGGVVVVLSSDSDDDHHHHHISFFFFIIRRIRRLLWWWMMMYYYYYGGAKKKKKQNNNNE
**3nzl-A**	AAEEQQQETTTTGGGGVVVVLSSDSDDDHHHHHISFFFFIIRRRMRLLWWWMMRYYYYYGGAKKKKKQNNNNE
	AAEEQQQETTTTGGGGVVVVLSSDSDDDHHHHLISFFFFIIRRRRRHLWWWMMMYYYYYGGAKKKKKQNNNNE
	AEAEEQQYYQGGFTTGVVVHVSSDSDDDLHHHHISFFFFIIRRRRRLWWWMMMLYYTTGGAKKKKKKNNNNQE
	AEAEEQQYYQGGGTGGVVVVLSSDSDDDLHHHHISFFFFIIRRRRWLWWWMMMYYYTTTGAKKKKKKNNNNQE
	AAQLQQVVVSSSFDDIDLHHHHGGGGGGLQATTTKKKKRRRRITNNFFNFYYYYYEEEEIMMMWWW
	AAQQGQVVVSSSFDDDIHHHHKKGGGGQLLATTTTKKKRRRRIFLNFNNFYYYYYEEEEIMMMWWW
	AAQQGQVVVSSSNDDDIHHHHKKGGGGQLLATTTTKKKRRRRIFLNFFNFYYYYYEEEEMMMIWWW
**3obh-A**	AAQQQAVVVSSSFDDIDHHHHKGGGGGQLLTTTTKKKKRRRRIFLNFNNFYYYYYEEEEIMMMWWW
	AAQQQFVVSSSVDDIDHHHHKGGGGGALLTTTTKKKKRRRRIFLNFNNFYYYYYEEEEIMMMWWW
	AAQQQKVVSSSFDDIDHHHHVGGGGGGLLATTTTKKKRRRRIFLNFNNFYYYYYEEEEIMMMWWW
	AAQQQVFVSSSFDDIDHHHHKGGGGGALLTTTTKKKKRRRRIVLNFNNFYYYYYEEEEIMMMWWW
	AAAQQQQVVVVSSSFDDDIHHHHKGFGGGGLLTTTTKKKKRRRRDFLNFNNFYYYYYEEEEIMMMWW
	AAAQQQQVVVVSSSFDDIDHHHHKGRGGGGLLTTTTKKKKRRRRIFLNFNNFYYYYYEEEEMMMNWW
	AAAQQQVVVFSSFFDDIDNHHHHAGGGGGGNSTTKKKKKRRRRRILLLFINFYYYYYEEETMMMWWW
**3obh-B**	AAAQQQVVVFSSFFDDIDNHHHHHGGGAGGNSTTKKKKKRRRRRILLLFINFYYYYYEEETMMMWWW
	AAAQQQVVVFSSFFDDIDNHHHHHGGGEGGNSTTKKKKKRRRRRILLLFINFYYYYYWEEETMMMWW
	AAAQQQVVVFSSFFDDIDNHHHHHGGGGGGNSTTKKAKKRRRRRILLLFINFYYYYYEEETMMMWWW
	AAAQQQVVVFSSFFDDIDNHHHHHGGGGGGNSTTKKKKKRRRRRILLLFINFEYYYYWEEETMMMWW
	RWWVMMMMVGGEEGVVYQTTTGGKKKKKQQYNNNEEAAAAQSDDDNSSHHHHHIITSFFFFYYYFRRRRRIDLLL
	VWWWMMMMVGGEEGVVYQTTTGGKKKKKQQYNNNEEAAAAQSDDDNSSHHHHHIITSFFFFYYYFRRRRRIDLLL
	VWWWMMMMVGGEEGVYYQTTTGGKKKKKQQYNNNEEAAAAQSDDDNSSHHHHHIITSFFFFYYLFRRRRRIDLLL
**5icb-A**	WSWMMMVVEGGGGGVVYQTTTGKKKKKKQQYNNNEEEAAAAQDDDNSSHHHHHIITWFFFFYYYFRRRRIIDLLL
	WVWVMMMMVGGEEGVVYQTTTGGKKKKKQQYNNNEEAAAAQSDDDNSSHHHHHIITSFFFFYYYFRRRRRIDLLL
	WVWWMMMMVGGEEGVVYQTTTGGKKKKKQQYNNNEEAAAAQSDDDNSSHHHHHIITSFFFFYYYFRRRRRIDLLL
	WWDVMMMMVGGEEGVVYQTTTGGKKKKKQQYNNNEEAAAAQSDDDNSSHHHHHIITSFFFFYYYFRRRRRIDLLL
	AAEEGGIMLYYQQWNNTTKKRRHHVFFDDS
	AAHHGGDTFFQQSSNNLKKKRREEVYYIWM
	AAHHGGIFTFQQSSNNLKKKRREEVYYDWM
**5znf-A**	AAHHGGTIFFQQSSNNLKKKRREEVYYDWM
	AKEEGGIMLYYQQWNNTKKARRHHVFFDDS
	AMEEGGIGWLYYQQNNTRRKKKHHVVFSSD
	AMKERFINNFYYSYGEGRTTKKLVHHQWSD
