# Supplementary material for: Transferable Coarse-Grained Potential for De Novo Protein Folding and Design
Source: PLoS One. 2014 Dec 1;9(12):e112852. doi: 10.1371/journal.pone.0112852 (PMC4249799; doi:10.1371/journal.pone.0112852)
Supplement: Table S5 — Sequences obtained during the last step of the matrix optimization procedure. The amino acid composition is identical for all sequences and the first is the natural sequences taken from the pdb file. (PDF) [file pone.0112852.s009.pdf]

TABLE S5: Sequences obtained during the last step of the matrix optimization procedure. The amino acid composition is identical for all sequences and the first is the natural sequences taken from the pdb file.

	GKITFYEDRGFQGHCYECSSDCPNLQPYFSRCNSIRVDSGCWMLYERPNYQGHQYFLRRGDYPDYQQWMGFNDSIRSCRLIPQHTGTFRMRIYERDDFRGQMSEITDDCPSLQDRFHLTEVHSLNVLEGSWVLYEMPSYRGRQYLLRPGEYRRYLDWGAMNAKVGSLRRVMDFY
	GRIQCGEDRYGQGMCNIYSMNYDPRMPERFRCRSCDDDIGLWEYYEQRNYRFHQLNLIRGRYPDQYGWCYSLYELVCRFLQPLNPSTSMMRRQYFDFRRGTVSEMTLLRPVIYDRFHPTGVHPQQKDWGDLVCDELVSYRGNKYLSTSQESSRFYSWGAGHAIHGFDRLDMFSE
	GLITMYYDRVFYSRCMLEDGQQELLIDHFSKCDSYYVPSHTWMMYEFLNYQGFQHFPTPGNEPNLQDYDSDNEQVLYCFYSCLHTGTARSRIFERDDFRGYQREIRSDCLRWSGRRRIKERASLGELCGSWVNQSMPYQGCRLYLPRPGDYIRRPDWGGMNHGVYSRQRVMDFR
**1AMM**	MYITFYEDRGYQGVCYECSSDKYGILVIGSRCYSYRVDSGTWMLSCVLQHQFHQYFFRRYSYPSYRQWMVMHCNDDPDQLPPGNTGRFRMRIEDRDDFRGLNSEIEDDRPSRQLSRHLTECHSQKGLEGFWNQYEMPLYRGCQYELTPGFDRLGLIWNARVARNGSLRRPMDFY
	GFYTFEEKRGFQPHCPPCSSDGVLLQPLFSRCNSIRVQSGRGVDYEEELYWYRQYFLRCMLYQDYGDKMLFYDSQTNCRNMPQHTYTFRMRIWEGDDWGRQMSGISDDCPSLQDRPHLQEPHSLNVCRTSGVLYEMFSYRGNIGLLRYGRYSRYIRIEAMNADRRDHRWVGDFY
	YPYIDHSWRGEQGFCCYGLSWMYLLQGGSQRCKFIRERLLCQMADDRPRDDQYWTMCSRSDLFGYQSWMGTDDRTRSFRRVPVHMSTFRSLIYNVRDSELIYQEIYFDVPQYQNYHDGTMVSFLNHHPDLGLGSEEYGCRGYQYGNRPSEYRFPLFNPARNIKRGERERVMDLC
	QRIDFGEDERRQSYNRFSSSRYPLRLFYMGRCNQIYWDRSCQMLIEFWNNIGHQYVLHRGDRNDYQMWEGIRDSKLSCGLIPNYMRTQRFRVRRRHCYDPQTSCLTYDCPSLSDLFYGWKVHSDRVLEGYTERAEQPSGPLTEPVLYPGRHMFFLDYDAMQYEGGSFVGGMDCY
	KSAKDALLLWCQMKTAGYPNVNIHNFTTSWRDGMAFNALIHKHRPDLIDFDKLKKSNAHYNLQNAFNLAEQHLGLTKLLDPEDISVDHPDEKSIITYVVTYYHYFSKM
	TSGADLIKLTINFKTDGYFKDDIKNKTMPARDNQVMKPAMALHRASLLSFQNLLLVNPEYNHCLWWFDADHHNLNFHDLHAYYKSVIKYEELSAPQDKHTKYGVTSII
	IKGKDALLHFCKNQTKIYPPHDGAKNIPSKRDAMKFMLLYHQWDETEKDADPGKKSNAFYTLQLDASLFLHHIENSNLDTIVRANHWDVIALLTLTYVVNYNHYFSSM
**1BKR**	TSGADLIKLTINFKTDGYFKHDIKMKAMPARDNQVKKPANALHKTSLISFQNLLLVNPEYDDCLWWFNHNHHYLDFHDLAAYNMSVIRYEELSAPQDKHTKYGVTSLI
	DSWKKALLYWHKNKTAGYPYKNHNNFTTSARLGDSLAAVIHFHNPMDFSLDKKNLSAAHLNLHLAFNLIEQQQGVTKLDDPEDIIVLHPMEKSIITYDVTYYMDFRKC
	ASASWKLLAWKQMASAGYKEVNHLLITTSIHDRMAFNNLIDKHERDLAYYDDKLKFNFPYTIQNEFIDDHQHYGKTCLLPPNLFSVDLPHAKSLITGVVTDNHYNKKM
	KSALDAILWWDDVKTMLYPNSNLHQFLVDLRPAAAFKALKYTHCPDKILFSKKKLSDKHDNLNNGHNRFEQHLGGYKYVNIEYISVDNPDETIQITTTLAMAHYFSHM
	MNDSEFHRLADQLWLTIEERLDDWDGDSDIDCEINGGVLTITFENGSKIIINRQEPLHQVWLATKQGGYHFDLKGDEWICDRSGETFWDLLEQAATQQAGETVSFR
	GTATERFHSSAVDGQFIQIRNLRWNLGDLDKCLIQETLWLLEKDFGLVIIIATWDHEDQDGTLDEATLIHNYGFEEEWMDQDSGEQSWCQGNPARKIDFEGTDSRV
	MTATEVFHSSAVDGGFIQGRNLRWQLHDLLKCEIQETDRLLLKDFGLVIIIATWDIEDQDGTLDEATLIHNYGFEEEWGDNDSGEQWWCQQNPARKIDFEGTDSSR
**1EW4**	MNQSWFHDDADGVWIFIEERLGDWDGDSDIDQEGNDGRLTITWEGSSTIIVNGQEQLWQVELFTKCDGYHFDLHGDEELCLLSANTFKRLLEQIATPQTIEKARAR
	SWCRQDMHLEDWWWLCLEALQLRNDILGDIFDALGDDSLGRHQKIETNIDSDVETPITAVLVQGDEINDNKTAQGGTEQEGHYQWTGIRFEAKLSTIFFDEERGFS
	ANDSDHWRLPSELLQTIEQFHDDLDGTSAYDQMINWTCFRETQEKGGIEIIERRDNIHLVWLFTKEWDGQLDIGGEEIVSDDCKQRLTVLLNQGADEAAGFTWSFG
	ANDSDHWRLPCELLQTIEQFLDDKDGDSAYDGQINWTVFRETQEKGGIEIINRWDMDHLVWLFTKFSVGCLDINGEEIIRDGSHERLGDLLEQAATQQAGETWSFT
	KQVEIFTDGSALGNPGPGGYGAILRYRGREKTFSAGYTRTTNNRMELMAAIVALEALKEHAEVILSTDSQYVRQGITQWIHNWKKRGWKTADKKPVKNVDLWQRLDAALGQHQIKWEWVKGHAGHPENERADELARAAAMNPTLEDTGYQVE
	WQYEHFTGHSGLGNPGPGGWLAIARYSKAEKDVRDTYTRTTDNNMPLMAANGAQEHLLHILGVLAETASAYVRQEPTKYIEIWQVVEGAGAMAKTVRKVDNWWRKDGLLTQKQIKNEWFQGHGDLAEIARKPERKRAAGKLNISEDTARQVE
	GQAETHTRDSGLHNRGWGVAISIPLYTHREKNATKDLKRTSENAVELMKGNVAYVAQKEDAEVLARTDMEYWRQGIQQLIYWWGPWGWGILDPSLMKADPALLRVFTALHAHVAKAEQGGGNAGGQENERIYEKRRPATTNKATEDKKFQVI
**1F21**	AGETEFVQTVAATNAGPYADGAIRALRGVIKTMKRGYLRIDKINIISMAKTHADEARDGVVKKKRHTEFNYLGQQILEGQQNWQSVYWWLKPDHYAANKSLWRDLLRVTGQLQGEDEWRHGTPLKPHEESAGENATAAALNPMWETRGGKEV
	KWYKMATLLLAKGTLGPGQYQARVQTNGTEKTFAVYIMVGTPHKRNLQAAIWAGEVETKHIQVPLDTDMIQEEEGRFRGIQYWVARKWKTGTGKVEKNWAAAGRLEWKLSANIREALHVSGHSDLDAHIDDDRYNRAAARNPNGESEGQLPE
	IQVDLFTDGSAIGKPNVGKLAAIDVGRWRRARFGAQWDARATNTMHLMAPRTAEGELNEKNTPIESGTSQEVAARIAEWIGYWKKYSWVGGGKGEVDVALTEGYLPRATGQHEIKWHLLKQHAREDHNQYQDKKKRAALMNPNLTETLYQVE
	KATGGFVEGLAASDDGMKLYIWNTIIRHDTWKFKAAYYTKTNNGEKQMDAGVAGTEGTEHLKVWESRLSQRVRQGIKRAHHNRIKEEVNLSDDQPWDNVILKPRQGAALMEQLITWKWEHPAPEAPRNTYVAERGRGGALLQLGAATEYQVA
	IDVSRLDLRIGCIITARKHPDADSLYVEEVDVGEIAPRTVVSGLVNHVPLEQMQNRMVILLCNLKPAKMRGVLSQAMVMCASSPEKIEILAPPNGSVPGDRITFDAFPGEPDKELNPKKKIWEQIQPDLHTNDECVATYKGVPFEVKGKGVCRAQTMSNSGIKL
	NDDSRPDYRIGTMSIAVVNEDADSLYVEEPKVIEVAPRLRVSLLVHQVPVEQMQNRMKCLPCLKKMAKDRHMLIDKMADCVSSTEKRTILAPLIGSVNGAGITFIVFPGECPKELVKKKLGWEQTQPPIHPNQEDIATLKGVPFENPGAGNCRAGVGSVSIIDL
	NDDSWPDYRIGDASIAVGNEDADSLYVEEPKVIEVAPRLRVSKLVHQVPVEQMQNRMKCLPCLKKMAKTRHMMIDKMADCVSSTEKRTDLAPNIGSVLGAGITFIVFPGECPKELVKKLLGREQTQPPIHKNQEIIATLPVVPFENPGLGNCRAGVGSVSIIDL
**1FL0**	NDDSWPDYRIGDASIAVGNEDAPSLHVEEPKIIEVAPRLRVSKLVHLVPVEQMQNRMKCGPCLKKMKATRYMMSDKMSDCVSSTEKRTDLADNIGTVLGAGITFIVFPGECPKELVKKLVGREQTQPPVHKNQEIIAILPVLPFENPGQGNCRAGVLAVSIIDL
	IDVSRAALETGKPGMNEGHLDFTSCYVTEVDVVVIPIRKRVSGLENTGQVEQGGQEMMIILLCNLLVAKMRMVWSQGKGRCASSPSKAEVLVPPIASVLGDPSTGDAHVNEPDKEINDIKKALIQLQPDPATKDICVAIIRGVPFEPKDRFLCRNPHMYNPKEKL
	IDVIRGALETGKPGMNEGHLDFTSCYVTEVDVVVIPICKRVFGLEITGQVEQGQEMMIILLCNLLVAKMRAVWSQGKARCGSSPSKAEVLVPPIASVLGDPSTADAHVNEPDKEINDSKKALNQLQPRVATKDIRVMIIRGPPFEPKDRSLCDNPHMYNPKEKL
	AEVPRVTLDIGEIITNVPMPDKQSLSVEELDVFEIARRSVVSRLDNHLLLEMMQNEHRILRSNRGAAPMGETPVQPMVAKARSKEYIPGLAPKNLSLPCDSPTFDMCPGAIDKDGAIKKKIWEQIQGGVHYNVICPVKVKGVPGCIKDCDVGLAQTFSNTEVKL
	RMLPRLCCLEKGPNGYGFHLHGEKGKLGQYIRLVEPGSPAEKAGLLAGDRLVEVNGENVEKETHQQVVSRIRAALNAVRLLVVDPETDEQL
	GLLQKEKSERNEMNERGVHLVGVGSKLALAIVLATVPGRERPEFACYLKCLPVLTGGPNLAEVHGKEYGALRLRQDIDGEDLVPHEQNVQR
	FVEDVHGVLGLQGGVNLGEPHPARGVLGKTICLANNPELYGPEGLYCRSLKGLKRNEDKEDEEREQVSRAAHILLVVMLLVKARPRTEQQA
**1G9O**	RMLTVLCCKELPFEGYDPHLHGETQVLGQLNRLYKPGLPAGKAGKKAGDREVEVNNENVSLEEHGQVSVRIRAALIAVRLLVRDPEGGEQL
	ILLPRLNPQEAGESPYVFGLVGEKGGLRRYLLAHEEHNNAEKKGVQKGDGLLLIPGECVDKMTPNQVVEGLQAVLRAARRLVHECETSDVR
	LMVHQLCNLLKNPGGQEVLALLCAPEYIQVPDNVGGASIAEKGDRGVRGLGVHKARLNLEEGTEHYAEKERRKQRVFVLLSVREPPTGEDL
	RNSPRVCEDEPGYKALLFHGHGVKKYVGNPILDEEVRTNAEGAGLVLGEHAVELPGENVRKKGRQQVLLRSGCELQAQPLLEVLITADRML
	GKITLYEDRGFQGRHYECSSDHPNLQPYLSRCNSARVDSGCWMLYEQPNYSGLQYFLRRGDYADHQQWMGLSDSVRSCRLIPHSGSHRIRLYEREDYRGQMIEFTEDCSCLQDRFRFNEIHSLNVLEGSWVLYELSNYRGRQYLLMPGDYRRYQDWGATNARVGSLRRVIDFS
	GGITNYMDRLFSLRGWEELRDHPRGQWMGRRCMRFRHDSGCWSCLEQINYSRLQYFLEPGDSSDVQEWYGASDQQRFCNLIPHYVSYRIGLYERSNIRSLRRQGTEDLSCLQHEFRSLELHSLNVRRCGPYLVVAMNYSYSFYYLSPKDGRHYQDSGATRQNVDLGIEYDDAR
	GHITRLERDGFRGRRIECYSQHSSQRYYRGRNHSCRADSYCRMNYMQPNRFGSQRFDGRGDSTDVWMLMSSNPSWWECQYYPHAQSHRINLYERWDVQGQAIESCPALFHLRYEEKFDFGRSDQILLVGVVDYGESNYELRTYLNSGGDLLRSRDYCDVLLEYLSLRLQGILP
**1HK0**	GRITRLERQGFRGRRIECYSQHENQRYYRGRSHSCRADSYCRMLYMQPNVFGSQRFDGRGDSTDVWMLMSSNPSWWSCQNYPHADSHRINLYERWDRQGQAIESCPALFHLRYEEKFDFIHSDQGLLVGVVDYGESNYELRTYLYSGGDLLRSRDYCGVNLEYLSLRLQDILP
	EPIWSYLSRGYIGLLEGCDFSHSDEQPYRNRCNSQEMNSGYWVHYECLLSYWCAPRRRHYRYTLSQTGDSLSDSVRLRRLPAHCDSHRIRRDEFQGGLDYHIEGVYFQSTLQCLFRFNGIMYRFVELEDRNLYEDQNQSRRQGLKMPGGYGRYQSWGALMDRVDALSRVIDNS
	GHLDILVYRQFPSPHCEHSSGLYDLHQPLSSCNRARVDSRSHSIYEQGNLEGLQRGIRTVDYERWQQWMGGSDLMRDCRLIRHPFDGRIKLYEDERDRQQMIECLCYCSSLGRSFWFNEYYRYNGLEFSRSLYALQNYRDGQYVLMPSNYDFYSDRWVTNARAGGGRRVLTES
	MEITLLECRIFQGESQEVSSDLSSFLPYLSLHNSARIDDGCWSKYLHPNLYGDQNFYYRWSYVRHQQGRRLSDSCRSCAHEPGSGEHRIRLYISERYRGQMLNFYYDCGDEQSDFDNLERRSMFGVCWPWVYYHRGNYRGMRYELQLGDTGLRQVRQATNDGLRPLRRVADIS
	TVAYIAIGSNLASPLEQVNAALKALGDIPESHILTVSSFYRTPPLGPQDQPDYLNAAVALETSLAPEELLNHTQRIELQQGRVRKAERWGPRTLDLDIMLFGNEVINTERLTVPHYDMKNRGFMLWPLFEIAPELVFPDGEMLRQILHTRAFDKLNKW
	TVAAMEGGGILLIPLELSNAALKRLRWKVDSFIILVPSFYPDPTNRRTHQPDYLNQHVTLTLILAPVELYAHNQRWEEQVPILAIAERTGPRSQSDDIMPFGNEEDNPARKRKLHWQLLLRVDMILPNFSEAGENVLPTGEMLDQLFATAAFEKLTGY
	TVAFIAIGSNQASPLEQLIAAKRAFGDIPENYNLAVSSFYRTPPLGPPDQQDYLNTAVALETSLAEEELFNHRARIELQQGRVRKLREWGPRTRDLTIMLVGNEFIPLSRQTVPHYAMKPKGFMLWPLHEIANELKLPDGEMDTLDLHVLDTIVLNLW
**1HKA**	TPEKFANLMDETAPLRLVNAALGALGRPPQSHINTVSIFYLKSELGRTFQPDYQWAAVTDERRKAPEEPNNHTHEIVLQQSGNRIAELLGNNTRDGDYMLITLPIGVPEQLSVPHYIMKRRDDMLWLLIEIAPALVFVDGEQLARSLETLAFFKPLLW
	APAYAIILSREFKLQLGANNAEKGPEHPQPSLENLDQSFYGTRLGHSQDLMLLPAAIEPMETIAAPELLLLDDVRILLQQSRVTSELTWGRVTGLLDIMLEVNEEINTTRVKVFHYDAKNWGPYLRFDAVIAPERPFPWNKMARQTLGIRLFDTPVNH
	EVAIIVIPSLLASPDDQTRATAVALLDPPEQHSLTVSSMLRTYALRSEDQPDDPRYALFLNFVLDPLTLLKEKQNYEHAQDTANGGEMWGPEVLNLRIMPWKNERIITARLKRPHYIGNNRGFFIVPLLHIAAELWFEPGGMLTQFLTERVQENLAKG
	RVQYVEASPPVASPLQRLNAPTAPLEGIPYFHITTLSSQVELPATLNQAQWDSLVLAGELRYRGDSEEGLAKLLTTTRFALRLRKAERMHPPILDQDIMANGIAKLTLFGVGLPQYHNNNNHFWLPPIKEVAVELIDEDDEMLNRILMIRFTDKGWFE

	LHLAFSSWLFNAKGQLLVTRRALSKKAWPGVWTNSVCGHPQLGESNEDAVIRRCRYELGVEITPPESIYPDFRYRATDPSGIVENEVCPVFAARTTSALQINDDEVMDYQWCDLADVLHGIDATPWAFSPWMVMQATNREARKRLSAFTQLKL
	LHLPFMKWDHNAPRTALSAVQALSWPPWFVNACNKICAEACLGEKDERWVDDTRTYEQGLEVDGADTRLPRTTYAGRGSSGHVECSAVTKFAASPFSLDQVPMIGVFVIQWQRFAYVLESYPVLPWNDSANMLLTKLNDEIRERLIIATQRSR
	AHVSFSSCIFNAKAQLDVEPRALSKGLSPGDWTNAAWGHPAAGESNEFARCRKCERELGQEITPREGIYPDWRVDATMPLKQLLNEFCQVFAARTTSLLVIKDDEVMDYYIWLLDLVLHAIPVTAFAVSSWMVLQQTNRTDYNRRPWGPVSRT
**1HZT**	LPAGFSLLTHLAKYCWMVKYGALNKEAWPDWFTNPHFGFVELVLATEDAVITRNREEQGSEIRPSSCTWPIFYMRAIPQFDEVSAVSCPVWAHRQTLAWSLVITENLDVPGCDLASYNAAIDQVPDNTKSDGSMLRRVRQARQREDRATGLKL
	PFLAFPFRLDSADAQALVTVLRESGVSWIGYWTNGSTISASEKLENELAVAREERYPIDAKLNPQLSDGCERYPVLTMFSGIKQMVVVPLCAARLTANLYQNLRSHMARQWFDNVWIPHADDVTPWTFEVRRAIPGLCRWGDKEHATQTSCKD
	TLLAAASSGTVIYGKLALTRKASVVESCPGVATYSFALHNQTRPSEWDFSIRRARCEDVVPCTLSVRNINEFRKRLVWRGKEPMNNDIPLQDSLTVDAPTGNDDQFMDYQWCDFHGVLLGILARPWAWLPFMHAIETPREAAYVLSAWQEQKE
	AASAFLSEEELDKGTFLVGNRLRSFDCDILSWRQSRPDTNQCGCLIGEAQSFELRYWVGVALTPPEFQYSALWERAMDVTGIVPNNVVPYIAANTVSTHQIPDIRKAMRPRDKCADVRVLWAMTPWAETPWKLLLENVDHARHTLSAFSQYKG
	HRQALGERLYPRVQAMQPAFASKITGMLLELSPAQLLLLLASEDSLRARVDEAMELIIAHG
	ARLLGAMLQTPMVLDRQLVAQQLHIDMFKEALARAPLSRSASYEEEAGRLHSLIELPILAG
	RPQALIERMSRFVQAMQPARAIALTGAKLELSVLDLLYGALSEDSLHARPGEAMELILLHQ
**1I2T**	RLAAVLERKYPTLQMMQLAESMVIIGHLEALFPILDLRGLASQDLLSGHQAEASEAARLRP
	HMPGLRARAAIVELFRLQMSLHLPMQSQAEEIKAGLLLGLLRRDSEPILVAAEAQTSLADY
	HDVILGLFLLEHPRAMQSPAAVLTRSMARELERLQIIALALRESGLLMLPYDKEQAGAQSA
	MPGSDASRELLAVLALAQMFDLQSLMEAAAESPHLLLRVLEIIRAQPQGRAHLGRKYLTEI
	AFDGTWKVDRNENYEKFMEKMGINVVKRKLGAHDNLKLTITQEGNKFTVKESSNFRNIDVVFELGVDFAYSLADGTELTGTWTMEGNKLVGKFKRVDNGKELIAVREISGNELIQTYTYEGVEAKRIFKKE
	NFDEFWGVDRGENTESVSMTTKGALVVRLFGIHSNIEYKEYGEENKKLVLFDVNSRTLFLMRWKKYKEKVNNAGDLKGEKIDTMVKGKNLEDIFGGDNQKNVIAEVTIAGVELKQTATYGFRTAKRIFETE
	LENGGAGEDLNSEYLRFMEEMFKNTVNLKLYVYLNRIFEIHQEMVNFADKATLRFDLKVAVIERETGKVTSTKNGTTGEITFSRSGKDVWKKVLKNDFKKGAGGNTEIVKREDGIQNAVKGVEWYDTEIKF
**1IFC**	NFDEFWDVGRGENTESVSMTTKGAAVVRLFGGGSNIEYKEYHEENKKLVLFDEDSRTLFLMRWKKYKEKVNNAGDTKGEKINLMDKIKNLEDIFGGVNQKNVIAAVTIEGVELKQTATYGFRTLKRIFETV
	VGDGTDKVKRNELDEKFRKTTFLNEVKMDLRAHDNLKLKITQTGNKFVVNESSNNNDIWVLTVFGVAFRFSLADKTALTGGWFMETNKLVGKIKRVENGKEIIAVRMISGKEEYQTYEYEGEEAGGIFKYE
	ALDGMDKVDRNENGITVEEKTNNNVEKRILEAWDNEELTNTQKGFKFTVKKWNIFRINDVVFGLGHDFAYSLAKKFTLTGTSTKEGNALVGMSKRVDSGYGLKEKAVIFGEVLIRTYEYMGVEQKRIFKEE
	AFIGIMVVNTTENVSLFIEKMGVDLKVTDLDKDVVKKWGFNQEGNENTLKGISTGRGVFVRFELGKYFAGSELTRTTLEGNKFMENVKGKYKITRKDADEEQEAIRVISNWKLKNNTEYEYAEALGRFHKD
	MTPAVTTYKLVINGKTLKGETTTKAVDAETAEKAFKQYANDNGVDGVWTYDDATKTFTVTE
	GADVTGTEDLVTADYELAKTEFKGGTETNTDNWTDPQKTNKVVKTAAIKVYAYTFTTAMKV
	YTTETTTPELETKFYKAQTKYTTDKGKVANNAFTGKINVAVDAATVAVMDETGGTLWDVDK
**1IGD**	NGGKAELVTTKTYKATAGDTKTVTVVEELFTVDNTKAYIMNTGDWKQTETAAYDFVTPDKA
	DTPTTTAYAKVKKDTNKTTKEYADKIDAATQWEEDVNMKFAAETNVVVTLFTVTYLGGGGT
	IEWTAGGYTLAAVDDTGEKNKTTTAKDAKTGYKEETNYLVKMTTTTVPQDDTFNAKFAVVV
	ANTAKAKYGFDLAVTMLTATKAFKYEEDETQTKAVVGTTINGGTPNVVDVKETTTTKWDDY
	KQVEIFTAGSALGNPGPGGYGAILRYRGREKTFSAGYTRTTNNRMELMAAIVALEALKEHAEVILSTDSQYVRQGITQWIHNWKKRGWKTADKKPVKNVDLWQRLDAALGQHQIKWEWVKGHAGHPENERADELARAAAMNPTLEDTGYQVE
	KQVEMQGAANRLSNKYKRPDGAIVHARGLGETFGAGKIATTNTPMTGMAQIVALLPTAEDAALIYEADDGKRHQSATQWDNKWGVRLQQTYINEPHNNVDAWQRGYAAFAEPVVEWSWLGLKKHGLEEGRVEWLAREIRLKGTIRHTEYKKS
	KQVALQGAANRLGNKYKRPDGAIVHARGLGETFSAGKIATTNTPMTGMAQIVALLETAEDAELILEKDDGKNHQSATQWDRKWGVRYQQTYINEPHNNVDPWQRGYAAFAEPVVLWSWEGLKAHGLEEGRVEWLARAIRMKGTIRHTEYKKS
**1JL1**	KQVRLQGAANRLGNKYKRPDGAIVHARGGGETFSAGEVATTNTPMALMEQIVALLATAEHAELILEKDDGKNHQSATQWDRNWGVRYQQTYINEPHKNVDPWQRGYAAKAEPVILWSWEGLKAGHLEEGRVEWLARAITMKGTIRDTKYFKS
	WLLLIATAYSALKMGKKGGNGQHVQTRGKMGEFRAIWTRQTKWSLALMAGGTTLEAVEVPIEEDQRTDWQRYHATIAYWKNSVEKKGENAANAIPVRGRILYLFAQDETNDGPIPKEAKSGHALHPDEERAGKLTRQAYNQARWNDVGVVHE
	KSKEADPHTGKLWMWRPLGYGYIGRYKGRTHPATSGALRTEYTGSQLAAVFAAKVRGEGKEVNILLGDSQYAAENEANTQAIIPKRHLEANNGRAVKNTDKWEREDLAQGQEATKNKWQMIHTDHMERLVTDALARIAVIWPVLEGQGWQVF
	KDEYLVTAGENLAIVKPDIGAQGEGAHNAWGGYSVKYTFKVYWQFELTAAIGLAERAKESAAEQENTATAYREKAIQQGIHGWWARTGTTDRVKISKSVALLQRTRDGLNQHAIKWEWPKTDMKVPRNHRMLNLARPQGLVPLNEDMGRGHE
	KEPEQLRKLFIGGLSFETTDESLRSHFEQWGTLTDCVVMRDPNTKRSRGFGFVTYATVEEVDAAMNARPHKVDGRVVEPKRAVSTVKKIFVGGIKEDTEEHHLRDYFEQYGKIEVIEIMTDRGSGKKRGFAFVTFDDHDSVDKIVIQKYHTVNGHNCEVRKAL
	ETPFTLDILDEKGLSFQTTETMLKSHDKQWGGLFPCNVMRDPCISKSRSFGFVRYATEDVYTAHSKMTKHGVTERVAGPIVNVHDVKKIVVEGLGTRNIELEHADYFGVKFRIEDIKDATGEFRGKKRGFAFVEKDDRVSRDEIEVQVYHHVTGENEQVRARK
	GAKKLQRKHMIQKMSPETPRESQWISHNDGGRLTDCLGRKVSVDTGSLGEGFRTDALKRVVFEAMNVNPFEPDIRVDVVKTRIRGIHKIFEEEKKHTDEAVYERHYFGDYVEIEEVGIFTSTVSGKKAGVAFDFFDDKENVDTTLGKTVFTVRYHECHVLQAR
**1L3K**	GKPESHRKGNNMSLGVPTDDRKDRGLVGVWRKLVGEIEAEVPVKIGSTRFAFHTFGTGEAIDNNMIDAPHKFDSRGGKKCVACQFIQKKFYHEVTTEEHVAELRDDIVQKGMREFKEIHTDRESVTKRDFAVVLRYDTTSETSYVIQLYGTVFVHEVEDRKFL
	KEDEQLAKLFIGILSKPTTDEQGFSHDGQWGTLTPCVVGVDENTKRFRRFLFVREATEEETDAANMARPHKTDERVVHPGRAVSTVDHIFVGEIKYDTEEKHLRDYFEQYSKIKVINGMTKRGSGGKRMSGRVVFDKHDSEDKIVIFYFHTVEGVNCVVRKAL
	VYVEQLGVLFRGRDHNAQKGIFIREHSTNWGTLMQFTGGHDPNHKRSFGFKTTTVMTAERVVKAVRRTVDKAYDNSRFPVAARKVVISIEVGGIDEDLFEHPKRTELKAQGKKEKICIMFGEDTYKERGPEFVTFDDHDEVELVVESKGHDVDSSRCEYTKIL
	KEPGHARKTEIKDLSETTTEVRLHVDVEQIGELTCCVDGRDMNDARSSGVGQWTAPTFELVRNTYTKKPDVVDGMVVEAKRVFSLVKKGGVGEIDEDYEFQKLRFQFFEYKKIAVHEAMTDRHHKGRRIFIFTNFVDGSSEDFGVIYKPHTVNGHLKRSRIAE
	SAYPITGKLGSELTMTDTVGQVVLGWKVSDLKSSTAVIPGYPVAGQVWEATATVNAIRGSVTPAVSQFNARTADGINYRVLWQAAGPDTISGATIPQGEQSTGKIYFDVTGPSPTIVAMNNGMEDLLIWEP
	SAYMITGKLGPETKAADLVGQVVLGWKVSDFKSSTAVEVGEMVASQVWENPATTNTNRGSVTPASSQTNIRTMGGITPRVDWQAAGVDPISTAGILQGEQSTGYIYFDVTGPDPTIVPAIIGAYLLLPWAN
	SAYEMRGTYGSDGLELDGVGIVPWDWYTISPPAVTAITVGKGAAEVVLEAIATPVGSVASGTGSNSQFTALTAFTNNLRVLWQAAGPPVDSGKTVNQQWQSTPKIKIDRVGPSTQYTTMNPIMEDALIVIG
**1LMI**	AIYSQTGLLDSTGYVEIVVAILVPDWPFKDELFKTAAIPIRPMQAMISTITQSVPQPGTSVTPGGAAVNAYEVEGANVMGQNVATSGTIVWQATKPTKESSIGTTNRDGSGARPWAVSDTWGLVDLNYGGL
	SPQPSALKLENRMGLTLTAGTTVNVIKWSGGYWGQAVFDDYPSDGEVQGATDAVVDGFGSYTPPGSVVLVESARIWNVTRSTIAWGQYTLSKANPPQGIQITAGISLEVTTPVGAITAMKNTMEADIPAVI
	TWYFIPPDVGVSWTGTNTGGLFVQDRKVSPLYGSVGDASTIIQTASVWESAAAGQLIAISGTLTYKIVNVRTAVPVNGISNWAADVDNTTDGQGGPKTELSYQEQIGPATGMRAASVAMIKPMEVPLTLEP
	NIYIDTWSLDTPLVMPSYRFDGNTVWEVSGIPSSTAVTPGTKVQGSGQKDGAIVNGARGKEILAVSPVTNTGAMVTASPESWAQVQAATTSTDYIRQGYAITGKVALAVVGPFPTIEADNGGMEQLLIWLP
	MDLSTKQKQHLKGLAHPLKPVVLLGSNGLTEGVLAEIEQALEHHELIKVKIATEDRETKTLIVEAIVRETGACNVQVIGKTLVLYRPTKERKISLPLE
	IELSGDMPQVLLLTTVTLKKLLLHHLTEHEVGLKPVVKIHIELEAKKLTDALAECRRVGEKQAEAIRVETSPQGRKNTGIVLPAYKIQTEIKNSVGLE
	HQLSTKVKTLLEGHAEELVLVTETLLIGAKVGKEALMTLILTVIEVKHNKRIPPDQGKSQIEVDKGEPEREACARPAIEKTTVHNRLVLLQKYSIGLL
**1LN4**	MRLLTLRLQHLKGKLHPLQKVVELGSNGLTIGVSNHIEQELEHEYLIKEKIATEDREVKTEETVAIVDETSACAVKVTAPTAVLKQPIKGRKIGLPLL
	ENLSTAKKLYLTVPRKIQLQERLAGETRPITIVKLETEQHLLLKAGIVVAVGSLQGHLKTKIEVELMIDVHHCDGVVIKKLLGPTRPKTEESENLAAE
	LLLVRAQEPITKMLEHEEKSVQVKTALKSKETKSEQHEAHIPVIIITTETGAALIEPKQLLYVPKIKTNLGACEGNDVRKEGVLRGLVLHRGTVLDLL
	LETRTDQKKLETKTGHPKLPVSTQLEAPVIEAVLGKGLGHAEVHSQVLERRAELDELNEYILSGVAIPETMACELTVIIKTLVLQKKKVIRNIKLGLH
