# Supplementary material for: Transferable Coarse-Grained Potential for De Novo Protein Folding and Design
Source: PLoS One. 2014 Dec 1;9(12):e112852. doi: 10.1371/journal.pone.0112852 (PMC4249799; doi:10.1371/journal.pone.0112852)
Supplement: Table S6 — Designed sequences under the additional constraint that local repetition of up to 5 residues are forbidden. (PDF) [file pone.0112852.s010.pdf]

TABLE S6: Designed sequences under the additional constraint that local repetition of up to 5 residues are forbidden.

	WDSMRTIKTRGEVYQLWNQMEAREALIKGNFGVFYHVYFHGKTRKAHQYDSND
	WDSMITRKTRGEVYQLWNQMEAREALIKGNFGVFYHVYFHGKTRKAHQYDSND
	WSDMLVKRTGKAVHYQSYNDTEIAERAIGNFGYQFHVGQFRKTRKLHYEWDNM
**1gab-A**	WDSMRTIKTRGEVYQLWNQMEAREALIKGNFGVFYHVYHFGKTRKAHQYDSND
	WDSMRTIKTRGEHFQLYNQMEAREALIKGNSGFVYHVYFHGKTRKAVYQSWND
	WSDMLVKRTGKAVHYQSYNDTEIAERAIKNFGYQFHVGQFRGTRKLHYEWDNM
	WDSMITRKTRGEVYQLWNQMEAREALIKGNFGVFYHVYHFGKTRKAHQYDSND
	QYWNEMRKTRKGYHVYHVAMTSWDGFSRISRILDFNHLIRDIHFNYGNYGQEKAEGQKVSWQHLVKTGEAMT
	QYWNEMRKTRKGYHVYHVAMTSWDGFSRISRILDFNHLIRDIHFNYFNYGQEKAEGQKASWQHLGKTGEAMT
	QYWNEMRKTRKGYHVYHVAMTSWDGFSRISRILDFNHLIRDIHFNYFNYGQEKAEGQKASWGHLVKTGEAMT
**1leb-A**	QYWNEMRKTRKGYHVYHVAMTSWDGFSRISRILDFNHLIRDIHFNYFNYGQEKAEGQKASEQHLVKTGEAMT
	QYWNEMRKTRKGYHVYHVAMTSWDGFSRISRILDFNHLIRDIHFNYFNYGQEKAEGQKVSWQHLGKTGEAMT
	QYWNEMRKTRKGYHVYHVAMTSWDGFSRISRFLDFNHLIRDIHFNYFNYGQEKAEGQKASWQHLVKTGEAMT
	NHWYFMKRITGRHVLHVLDSTWSDLVQGETGKQSYQVEAKMTKGYFGYHFGMKIRFIRANWDQEARLSNRID
	EKAEGQNYGQAKRAKRSQNDFGVHLRISRISDILHDVFHVSYGLTKGTEAVYQWYGNMTKERFYWFNWMHI
	EKAEGQNYGQAKRAKRSQNDFLVHLRISRISDILHDVFHVSYGLTKGTEAVYQWYGNMTKERFYWFNWLHI
	EKAEGQNYGQAKRAKRSQNDFLVHLRISRISDILHDVFHVSYGLTKGTEAVYQWYGNMTKERFYWFNWKHI
**1pou-A**	EGANGQNYGQEKGAKRTNDWFHVLHRISRISDILHDVFLVSQGSTGAKEAVQYWLGKMTKERFYWFYNMHI
	EKAEGQNYGQAKRAKRSQNDFLVHLRISRISDILHDVFHVSYGLTKGTEAVYQWYGNMTKERFYWFNWMHI
	EKAEGQNYGQAKRAKRSQNDFLVHLRHSRISDILHDVFHVSYGLTKGTEAVYQWYGNMTKERFYWFNWMHI
	EKAEGQNYGQAKRAKRSQNDFLVHLRISRISDILHDVFHVSYGLTKGTEAVYQWYGNMTKERFYWFNEMHI
	DHMDSWHKMLVYFVYFHGRITRIMKGRTSNQEANTWNQDEALKGRHGYFVYFLKQAE
	SDWHSWTKMLVYFVYFHRGITRIMKGRTGNQAENDSNEDAQLKGRHGYFVYFLKQAE
	SDWMSWTKMLVYFVYFHGRITRIMKGRTGNQSENADNEDAQLKGRHGYFVYFLKQAE
**1qyp-A**	SDWISWHKMLVYFVYFHGRITKRMKGRTGNQSENDANEDAQLTGRHGYFVYFLKAEQ
	DIMDSWHKMLVYFVYHFGTRIGMRKGRTSNQEADNWSQENALKGRFGYHVYFQKLAE
	SIMDSWHKILVYFVYHFGTRITMRKGRTGNEAQNWDNAEKALKGRFGYHVYFLQSEQ
	SDMHSWTKMLVYFVYFHGRIMTRIKGRTGNQAEDNWSNQAELKGRHGYFVYFLKQAE
	WSDWLHMIRTGRKMTNEQAEKTGRHVLMVHFYGQEAQKNDSNQLNKEAKGYQVYFGYFRIFRISDWIHVLFYGHAK
	WSDWLHMIRTGRKMTNEQLNKTGRHVLMVHFYGQERQKNDSNQAEKAEKGYQVYFGYFRITRISDWIHVLFYGHAK
	WSDWLHMIRTGRKTFNEQLNKTGRHVLMVHFYGQEAQKNDSNQAEKAEKGYQVYFGYFIRTIRWSVIMVLFYGHAK
**1sro-A**	WSDWLHMIRTGRKTFNEQLNKTGRHVLMVHFYGQEAQKNLSNQAEKAEKGYQVYFGYFIRTIRDWSIMVLFYGHAK
	WSDWLHMIRTGRKMTNEQAEKTGRHVLMVHFYGQEAQKNDSNQLAKEAKGYQVYFGYFRITRISDWIHVLFYGHAK
	WSDRLHMIRTGRKMTNEQAEKTGRHVLMVHFYGQEAQKNDSNQLNKEAKGYQVYFGYFRITRISDWIHVLFYGHAK
	WSDWLHMIRTGRKTFNEQDNKTGRHVLMVHFYGQEAQKNDSNQAEKAEKGYQVYFGYFIRTIRWSDIMVLFYGHAK

	IRTINYHFYGFKGTRKTGNYGQEYKAEKAEQNWSNLAKEAVGQWYQVTMGLDVFHVRHSRIDLFDIMSHW
	IRKMNYHFYGFKGTRKTGNYGQEYKAEKAENQWSNLAKEAVGQWYQVTMGDLVFHVRHSRIDLFDIMSHW
	IRKMNYHFYGFKGTRKTGNYGQEYKAEKAEQNWSNLAKEAVGQWYQVTMGDLVFHVRHSRIDLFDIMSHW
**1utg-A**	IRTMNYHFYGFKGTRKTGNYGQEYKAEKAENQWSNLAKEAVGQWYQVKMGDLVFHVRHSRIDLFDIMSHW
	IRTMNYHFYGFKGTRKTGNYGQEYKAEKAENQWSNLAKEAVGQWYQVTLGDLVFHVRHSRIDLFDIMSHW
	IRTMNYHFYGFKGTRKTGNYGQEYKAEKAENQWSNLAKEAVGQWYQVTMGDLVFHVRHSRIDLFDLMSHW
	ILMHQVYFGTRKGRHKYVQFGQSGKLTKAVSNWSQEAVKALGTWGFDRMTIRYHLYFDNMIAERHENDWN
	VEGSTGKTLKAHVQYFGYRFKRHIRHNIRKILNFYSQGFAKGTSQYVLMWDEWDNMDAE
	VEGSTGKTLKAHQVYFGYRFKRHIRHNIRKILNFYSQGFAKGTSQYVLMWDEWDNMDAE
	VEGSTGKTLKAHVQYFGYRFKRHIRHNIRKILNFYSQGFAKGTSQYVLMNDWEDAMEDW
**1uxd-A**	VEGSTGKTLKAHVQYFGYRFKRHIRHNIRKILNFYSQGFAKGTSQYVLMWDEWDNMWAE
	VEGSTGKTLKAHVQYFGYRFKRHIRHNIRKILNFYSQGFAKGTSQYVLMWDEWANMWDE
	VEGSTGKTLKAHVQYFGYRFKRHIRHNIRKILNFYSQGFAKGTSQYVLMNDWEADMWDE
	VEGSTGKTLKAHVQYFGYRFKRHIRHNIRKILNFYSQGFAKGTSQYVLMWDEWDNMADE
	DNWSQRKTRKITKRAEMWQYGFYGFHVLDSWQSDAVHFGYFHEGMIKTRKALVFLGNHYE
	DNWSQRKTRKITKRAEMWQYGFYGFHVLDSWQSDAVHFGYFHEAGMIKTRKLVNLGNHYE
	DNWSQRKTIKRGKRAEMWQYGFYGFHVLDQEWSDAVHFGYHFETAIMKTRKLVNLGNHYQ
**1vif-A**	DNWSQRKTRKIGKRAEMWQYGFYGFHVLDSWQSDAVHFGYHFEITMRKTRALVNLGNHYE
	DTWSQRKTRKIGKRAEMWQYGFYGFHVLDSWQSDAVHQGYFHEATMIKTRKLVNLGNFYE
	DNWSQRKTRKIGKRAEMWQYGFYGFHVLDSWQSDAVHQGYFHEMTIKTRKFLVNLGNFYE
	DNWSQRKTRKIGKRAEMWQYGFYGFHQLDSWQSDAVHFGYFHEATMIKTRKLVNLGNHYE
	IRGHRKTMVYFLDMSDWHLYHFGKAEKAEQNYQNYGFHVIRTSWQSDNEAKGFYLRITRG
	IRGHRKTMVFYLDMSDWHFVHFGKAEKAEQNYQNYGFHVIRTSWQSDNEAKGFYLRITRG
	IRGHRKTMVYFLDMSDWHLVHFGKAEKAEQNYQNYGFHVIRTSWQSWNEAKGFYLRKTRG
**2cdx-A**	IRGHRKTMVYFLMDSWDHLVHFGKAEKAEQNYQNYGFHVIRTKWQSWNEAKGFYLRITRG
	IRGHRKTMVYFLDMSWDHRVHFGKAEKAEQNYQNYGFHVIRTSWQSDNEAKGFYLRITRG
	IRGHRKTMVFYLDMSWDHLVHFGKAEKAEQNYQNYGFHVIRTSNQSWNEAKGFYLRITRG
	IRGHRKTMVFYLDMSDWHLYHFGKAEKAEQNYQNYGFHVIRTSWQSWNEAKGFYLRITRG
	DLIRFYGTKAEVANSQWYQNMKGHFRIHLWHFMRKVYQKEALENSQKGTYGHYVSTGNVGKRFIRHIMRFYGTKAENLDWQDWMDAE
	DLIRFYGTKAEVANDQWYQNMKGHFMIHLWHFRTKVYQSEALENSQKGTYGHYVSTGKVGKRFIRHIMRFYGTKAENLDWQSWRDAE
	DLIRFYGTKAEQANDQWYQNMTGHFMIHLWHFRTKVYQSEALENQSKGFYGHYVSTGKVGKRIFRIHMRFYGTKAENLDWQSWMDAE
**2kyw-A**	DLIRFYGTKAEVANDQWYQNMKGHFMIHLWHFRTKVYQSEALENSQKGTYGHYVSTGKVGKRFIRHIMRFYGTKEANLDWQSWMDAE
	DLIRFYGTKAEVANDQWYQNMTGHFRIHLWHFMRKVYQSEALENSQKGTYGHYVSTGKVGKRFIRHIMRFYGTKAENLEWQSWMDAE
	DLIRFYGTKAEVANDQWYQNMTGHFMIHLWHFRTKVYQSEALENQSKGFYGHYVSTGKVGKRFIRHIMRFYGTKAENLDAQSWMDAE
	DLIRFYGTKAEVANDQWYQNMKGHFMIHLWHFRTKVYQSEALENQSKGTYGHYFSTGKVGKRFIRHIMRFYGTKAENLDWQSWMDAE

	WDSIRTIMFKYHFYHFGRIKRMFNYGFYGVHRTSIRDLFHVLHDVSDQSVTLKGEAGQNYQEKAGTKRAEWDNGVQYGKTANQWEALMNSWE
	SEYVQYGVFRTKGRTGFYVFYWGHMRKTRAKGQYGKNTGKEAKEQAEQANLDNLDNRISRILHFIDHFLDTMSDQHRFYHLMISWEAVMNSW
	WDSIRTIMFKYHFYHFGRIKRMFNYGFYGVHRTSIRDLFHVLHDVSDQSVTLKGEAGQNYQEKAGTKRAEWDNMVQYGKTGNQWEALMNSWE
**2kzv-A**	SEYVQYGVFRTKGRTGFYVFYWGHMRKTRAKGQYGKNTGKEAKEQAEQANLDNLDNRIARILHFIDHFLDTMSDQHRFYHLMISWEIVMNSW
	WDSIRTIMFKYHFYHFGRIKRMFNYGFYGVHRTSIRDLFHVLHDVSDQSVTLKGEKGQNYQEKAGTKRAEWDNMVQYGKTANQWEALMNSWE
	WDSIRTIMFKYHFYHFGRIKRMFNYGFYGVHRTSIRLDFHVLHDVSDQSVTLKGEAGQNYQEKAGTKRAEWDNMVQYGKTGNQWEALMNSWE
	WDSIRTIMFGYHFYHFGRIKRMTNYGFYQVHFRSIRDIFHVLHVLDSQWDNLTKEAGEQYLGKTGNAKEASWNEKQYGKRTGQMVAEWSNMD
	WHRIKTLSGFQVYQVKAGNEGAEKGMNFYLFYRHIMDIFRHGKHGKRTQSYQNVLAEDWSDM
	WGQVYQSMGTNKFHERIMDIYFHRGTRGKLGKVWQSTAVGAKEGFYQFYNLIHAERDNMDLW
	WHRIKTLSGFQVYQVKATNEGAEKGTNFYLFYRHIMDIFRHGKHGKRTQSYQNLVAEDWSDM
**2l09-A**	WHRIKTLSGFQVYQVKATNEGAEKGMNFYLFYRHIMDIFRHGKHGKRTQSYQNVHAEDWSDM
	WHRIKTLSGFQVYQVKATNEGAEKGMNFYLFYRHIKDIFRHGKHGKRTQSYQNLVAEDWSDM
	WHRIKTLSGFQVYQVKATNEGAEKGMNFYLFYRHIMDIFRHGKHGKRTQSYQNVLAEGWSDM
	WGQVYQSMKTNKGHERIMDFYLHRGTRGTLKGVWQSTEVGAKEGFYQFYNLHIARISDWNDA
	MWAEMNSWEAVMNSWQEVAKGTFRHILDNIHLYFQGKATKVYQGYQGRTKRHLFDNSDRIHLYFVVYQSTGKTGFHRID
	SWDAMNSWEAVMNSWQEAKTGYFRIHLNDLIHFYQGKEQVYQVGKTGRTKRHVFLDSIDHFNYMKAEWGTKGYFRIHR
	MWAEMNSWEAVMNSWQEVAKGTFRHILDNIHRYFQGKATKVYQGYQGRTKRHLFDNSDRIHLYFVVYQSEGKTGFHRID
**2ptl-A**	MWAEMNSWEAVMNSWQEVAKGTFRHILDNIHLYFQGKATKVYQGYQGRTKRHLFYNSDRIHLYFVVYQSEGKTGFHRID
	MWAEMNSWEAVMNSWQEVAKGTFRHILDNIHLYFQGKATKVYQGYQGRTKRHLFDNHDRIHLYFVVYQSEGKTGFHRID
	MWAEMNSWEAVMNSWQEVAKGTFRHILDNRHLYFQGKATKVYQGYQGRTKRHLFDNSDRIHLYFVVYQSEGKTGFHRID
	MWAEMNSWEAVMNSWQEVAKGTFRHIFDNIHLYFQGKETKVYQGYQGRAKRDNFDHLRIDHLYFVVYQSTGKTFRHIRS
	SWQSGKVFYIHRIDMIRYFVGTKLANELAKVGHRIKGLFYVQSWQDNEANTGRTIRHWDHLYFVGYQAENWMDNEYQKGHFMTSRGTKAE
	SWQSGTVFYIHRIDMIRYFVGTKLANELAKVGHRIKMLFYVQSWQDNEANKGRTIRHWYHLYFVGYQAENWMDNEYQKGHFMTSRGTKAE
	SWQSGKVFYIHRIDMIRYFVGTKLANELAKVGHRITKLFYVQSWQDNEANKGRTIRHWDHLYFVGYQAENWMDNEYQKGHFMTSRGTKAE
**3mx7-A**	SWQSGKVFYLHRIDMIRYFVGTKLANELAKVGHRIKMLFYVQSWQDNEANTGRTIRHWDHLYFVGYQAENWMDNEYQKGHFRTSRGTKAE
	SWQSGKVFYIHRIDMIRYFVGTKLANELAKVGHRIKMGFYVQSWQDNEANTGRTIRHWDHLYFVGYQAENWMDNEYQKGHFMTSRGTKAE
	SWQSGKVFYIHRIDMIRYFVGTKLANELAKVGHRIKMLFYVQSWQDNEANKGRTIRHWDHLYFVGYQAENWMDNEYQKGHFMTSRGTKAE
	SGQSGKVFYIHRIDMIRYFVGTKLANELAKVGHRIKMLFYVQSWQDNEANTGRTIRHWDHLYFVGYQAENWMDNEYQKGHFMTSRGTKAE
	MNLWEIREAIMLNYWQHEARKTNFYQHGKRTKGFVYFQGSTRKALVHDGSD
	MNYWEIREAIMLNYWDHEARKTNFYQHGKRTGKFVYFQGSTRKALVHQWSD
	MNYWEIREAIMNLYWQHEARKTNFYQHGKRTGKFVYFQGSTRKALVHDWSD
**3nmd-E**	MNLWEIREAIMLNYWQHEARKTNFYQHGKRTGKFVYFQGSTRKALVHDQSD
	MNYWEIREAIMLNWYQHEARKTNFYQHGKRTGKFVYFQGSTRKALVHDWSD
	MNYWEIREAIMLNYWQHEARKTFNYQHGKRTGKFVYFQGSTRKALVHDWSD
	MNIWEYREAIMLNYWQHEARKTNFYQHGKRTGKFVYFQGSTRKALVHDWSD

	QYEQGYFGHMTIRFYGKETNYEKGMYFHIDRSTLKAVQNWGSDQSRLIDWFHLVHAVDSWRTKNGEKVA
	HGTKGTEGLTMAQEGKTGSQKVAKVQYNLDNHYFRKIMSHRIFRIKDEWSNEARILHWNDMYQVYFGYF
	HGTKGTEGLTMAQEGKTGSWQVAKVQYNKDNHYFRKIMSHRIFRIKDEWSNEARILHWNDMYQVYFGYF
**3nrl-A**	SGTKGTEGALIAQEGKTVQWSQVKLQYNLMNHFYHKRDSTRIFRIKREWDNEARIFHWNDMYQVYHGYF
	HGTKGTEGLTMAQEGKTGSWQVAKVQYNLDNHYFRKIMSHRIFRIKDEWSNEARILHWNDLYQVYFGYF
	HGTKGTEGLTMAQEGKTGFQWVAKVQYNLDNHYFRKIMSHRIFRIKDEWSNEARILHWNDMYQVYFGYF
	SGTKGTEGALMAQEGKTVQWSQVKLQYNLMNHFYHKRDSYRIFRIKREWDNEARIFHWNDMYQVYHGYF
	IHLWFRIMYLFYVQHGKTGKAVYQWSNEKAEKAENFYGQNSGQNAVEDSWDSLVKTRMTGRHGFHYFRITRMD
	MHLWHRIMYLFYVQHGKTGKDVYQWSNEKAEKAENFYGQNSGQNAVEASDWSLVKTRMTGRHGFHYFRITRID
	LHMWFRIMYLFYVQHGKTGKAVYQWSNEKAEKAENFYGQNSGQNAVEDSWDSLVKTMRTGRHGFHYFRITRID
**3nzl-A**	MHLWFRIMYLFYVQHGKTGKDVYQWSNEKAEKAENFYGQNDGQNAVEASDWSLVKTRMTGRHGFHYFRITRID
	MHLWFRIMYLFYVQHGKTGKDVYQWSNEKAEKAENFYGQNWGQNAVEASDWSLVKTRMTGRHGFHYFRITRID
	MHLWHRIMYLFYVQHGKTGKAVYQWSNEKAEKAENFYGQNSGQNAVEDSWDSLVKTRMTGRHGFHYFRITRID
	WHLWIMRIFLYFGVHGKTRKSVYQDSNEKAEKAENFYGQNSQGNAVEALDWSQVKTMRKGRHYFHYFIRTIMD
	WLIRGHRKTGNYMQNWQEAKEGFYVFIRMIDLFHVFKGEKAENYWQYNQTKHRSTDSGDLVHAVTS
	WLRIGHRKTGNYMQNWQEAKEGFYVFIRMIDLFHVFKGEKAENYWQYNQTKHRSTDSGDLVHAVTS
	WLIRGHRKTGNYTQNWQEAKEGFYVFIRMIDLFHVFKGEKAENYWQYNQTKHRSTDSGDLVHAVMS
**3obh-A**	WLRIGHRKTGNYTQNWQEAKEGFYVFIRMIDLFHVFKGEKAENYWQYNQTKHRSTDSGDLVHAVMS
	WLIRGHRKTGNYMQNWQEKAEGFYVFIRMIDLFHVFKGEKAENYWQYNQTKHRSTDSGDLVHAVTS
	WLIRGHRKTGNYTQNWQEAKEGFYVFIRMIDLFHVFKGEKAENYWQYNQMKHRSTDSGDLVHAVTS
	WLRIGHRKTGNYMQNWQEKAEGFYVFIRMIDLFHVFKGEKAENYWQYNQTKHRSTDSGDLVHAVTS
	HWLMIRGHRKTGNYTQNWQEAKEGFYVFHRIFDLFHVYKGEKAENYWQYNQMKTRSTDSGDLVHAVS
	WHLMIRGIRKTGNYMQNWQEAKEGFYVFHRVIDLFHVYKGEKAENYWQYNQGKTRSTDSGDLVHAVS
	WHLMIRGIRKTGNYMQNWQEAKEGFYVFHRSIDLFHVYKGEKAENYWQYNQGKTRSTDSGDLVHAVS
**3obh-B**	WHLMIRGIRKTGNYMQNWQEAKEGFYVFHRIMDLFHVYKGEKAENYWQYNQGKTRSTDSGDLVHAVS
	WHLMIRGIRKTGNYMQNWQEAKEGFYVFHRMIDLFHVYKGEKAENYWQYNQGKTRSTDSGDLVHAQS
	WHLMIRGIRKTGNYMQNWQEAKEGFYVFHRMIDLFHVYKGEKAENYWQYNQGKTRSTDSGDLVHRVS
	WHLMIRGIRKTGNYMQNWQEAKEGFYVFHRMIDLFHVYKGEKAENYWQYNQGKTRSTDSGDLVHQVS
	AQESNAKEAKYQGYNGTMKAEKGYFVGKRTKRSWQDVLHVDSMDLHWLHRTIRTIGMHVYFGEQFYNLFNDIRSI
	AQESNAKEAKYQGYNGTMKAEKGYFVGKRTKRSWQDVLHVDSIDLHWLHRTIRTMWHGVYFGEQFYNLFNDIRSI
	EQANEAKEAGYQGYFGTAKMTKGYFVGKRTKRSWQDVLHVDSMDLHWLHRMIRTIWHFVYRGEQFYNLFNDIRSI
**5icb-A**	EQANEAKNAKYQGYEGTMKATKGYFVGKRTKRSWQDVLHVDSMDLHWFHRMIRTIWHFVYRGEQFYNLFNDIRSI
	EQANEAKEAGYQGYNGTAKMTKGYFVGKRTKRSWQDVLHVDSMDLHWFHRMIRTIWHFVYRGEQFYNLFNDIRSI
	AQSNEAKEAKYQGYNGTMKAEKGYFVGKRTKRSWQDVLHVDSMDLHWLHRTIRTFWMHGYFGEQHYNLFNDIRSI
	QSANEAKEAKYQGYNGTMKAEKGYFVGKRTKRSWQDVLHVDSMDLHGWHRTIRTIWMHVYFGEQFYNLFNDIRSH

	MKTRGFNVHYGFEGHLYKLIKRAERSQWND
	MKTIGHNVFYGFEYHQYKLTKRAERSQDWN
	MTKIGSNFVYHGEYRQYKNTKRAERHQWDL
**5znf-A**	MTIRGSDFVYHGEFGQYKLTKRAERHQWDN
	MTIRGSNFVYHGEFGQYKLTKRAERHQWDY
	MTKIGSNFVYFGEHFQYGLTKRAERHQWND
	MKTRGFNVHYGFEGHQYKLIRKAERSQWND
