# Supplementary material for: Transferable Coarse-Grained Potential for De Novo Protein Folding and Design
Source: PLoS One. 2014 Dec 1;9(12):e112852. doi: 10.1371/journal.pone.0112852 (PMC4249799; doi:10.1371/journal.pone.0112852)
Supplement: Table S7 — Summary of the refolded structures with the natural sequences. The DRMSD value is taken form the minimum of the folding free energy (see Fig. 5), while the Overlap, the gRMSD and the TM-score are calculated using the Max Cluster program from Alex Herbert (http://www.sbg.bio.ic.ac.uk/maxcluster/index.html). The Overlap is a measure of percentage of matched structural elements between the native and the refolded structures. The gRMSD and TM-score are calculated over the overlapping structural elements. TM-score was defined by Zhang et al. [48], [49]. The low overlapping value for 2ptl and 1vif are due to the unstructured sections of the proteins. (PDF) [file pone.0112852.s011.pdf]

TABLE S7: Summary of the refolded structures with the natural sequences. The DRMSD value is taken form the minimum of the folding free energy (see Fig. 5), while the Overlap, the gRMSD and the TM-score are calculated using the Max Cluster program from Alex Herbert[a]. The Overlap is a measure of percentage of matched structural elements between the native and the refolded structures. The gRMSD and TM-score are calculated over the overlapping structural elements. TM-score was defined by Zhang et al. [7, 8]. The low overlapping value for 2ptl and 1vif are due to the unstructured sections of the proteins.

PDB id	DRMSD	MaxCluster Overlap	gRMSD	TM-score
2kyw	1.75000	0.804598	3.499	0.505
2kzv	1.75000	0.554348	5.719	0.274
2l09	1.85000	0.887097	3.559	0.477
3mx7	1.80000	0.488889	4.773	0.261
3nmd	0.20000	1	4.428	0.423
3obh-A	1.40000	0.893939	6.661	0.512
3obh-B	1.75000	0.895522	4.532	0.482
1gab	1.60000	0.943396	3.032	0.542
1leb	1.90000	0.888889	4.048	0.486
1pou	1.85000	1	3.236	0.569
1sro	1.90000	0.526316	4.525	0.264
1utg	1.90000	0.8	4.146	0.434
1uxd	1.75000	0.627119	5.229	0.240
1vif	1.80000	0.4	4.496	0.206
2ptl	2.10000	0.371795	4.273	0.223
5icb	1.75000	0.986667	3.351	0.550

[a]http://www.sbg.bio.ic.ac.uk/ maxcluster/index.html
