# Supplementary material for: Transferable Coarse-Grained Potential for De Novo Protein Folding and Design
Source: PLoS One. 2014 Dec 1;9(12):e112852. doi: 10.1371/journal.pone.0112852 (PMC4249799; doi:10.1371/journal.pone.0112852)
Supplement: Text S1 — Supplemental Material containing the details about the model and simulations techniques together with the derivation of the scoring function with the Maximum Entropy Principle. (PDF) [file pone.0112852.s012.pdf]

Supplemental Material for Transferable coarse-grained potential for *de novo* protein folding and design

Ivan Coluzza[1,*]

[1]*Faculty of Physics, University of Vienna,*
Boltzmanngasse 5, 1090 Vienna, Austria

Details of the Caterpillar interaction potential

The Caterpillar model [1] is a 5-atom model of the protein backbone (see Fig. 1). The degrees of freedom of the model are the torsional angles ϕ_1 and ϕ_2; all other structural parameters are kept fixed at values from the literature [2]. Backbone hydrogen bonds are modeled with a 10-12 Lennard-Jones type potential using the expression [3]

$$E_H = -\epsilon_H \left(\cos\theta_1 \cos\theta_2\right)^{\nu} \left[5 \left(\frac{\sigma}{r_{\mathrm{OH}}}\right)^{12} - 6 \left(\frac{\sigma}{r_{\mathrm{OH}}}\right)^{10} \right], \tag{S1}$$

where r_{OH} is the distance between the hydrogen atom of the amide group (NH) and the oxygen atom of the carboxyl group (CO) of the main chain. We set $\sigma = 2.0$ Å, $\epsilon_H = -3.1\ k_B T_{\mathrm{Ref}}$, and $\nu = 2$ [3]. The side chain interactions are represented by and effective C_α-C_α sphere-sphere interaction energy given by

$$E_{ij}\left(r_{ij}\right) = \epsilon_{ij}\Gamma(r_{ij}) = \epsilon_{ij}\frac{1}{1 + \mathrm{e}^{-(r_{\max} - r_{ij})/W}} \tag{S2}$$

where $W = 0.4$Å, r_{ij} is the distance between the C_α atoms at the centres of spheres i and j and $r_{\max}$ ($r_{\max} = 12$ Å) is the distance at which $E_{ij} = \epsilon_{ij}/2$. The ϵ_{ij} are the elements a 20 by 20 matrix each defining the strength of the interaction of each type of amino acid with the others (see Tab. S1 for the values optimized with the MEP).

The residue-solvent interaction is modelled as a simple energy penalty towards surfaces exposure of hydrophobic amino acids; the expression has the form

$$E_{\mathrm{Sol}}(\Omega - \Omega_i) = \begin{cases} \epsilon^i_{\mathrm{Sol}}\left[\Omega - \Omega_i\right] & \Omega_i \lesseqgtr \Omega \quad \epsilon^i_{\mathrm{Sol}} \gtrless 0 \\ 0 & \Omega_i \gtrless \Omega \quad \epsilon^i_{\mathrm{Sol}} \gtrless 0 \end{cases}, \tag{S3}$$
$$\Omega_i = \textstyle\sum_j \Gamma(r_{ij})$$

where $\Gamma(r_{ij})$ is given in Eq. (S2), Ω is the threshold for the number of contacts in the native structure above which the amino acid is considered to be fully buried and the $\epsilon^i_{\mathrm{Sol}}$ are taken from the the Dolittle hydrophobicity index [4] and are positive for hydrophobic amino acids and negative of the hydrophilic ones. The interaction penalises the exposure (burying) of hydrophobic (hydrophilic) residues above Ω. The formation of sulphur bridges as well as Proline rigid bonds is not included. The total energy of a protein E is then given by:

$$E = E_H + \alpha E(\epsilon, E_{\mathrm{HOH}}, \Omega)$$
$$E(\epsilon, E_{\mathrm{HOH}}, \Omega) = \sum_{kl}^{N} \epsilon_{kl}\Gamma(r_{kl}) + E_{\mathrm{HOH}} \sum_{k}^{N} E_{\mathrm{Sol}}(\Omega - \Omega_k) \tag{S4}$$

where E_H in Eq. S1 is the total energy of the backbone hydrogen bonds, the α scaling factor is necessary to balance the two contributions to the total energy. Large values of α will tend to break the maximum valence principle, while small values will over-favour the hydrogen bond term inducing the formation of just helices. Finally E_{HOH}, like α, rescales the Dolittle hydrophobicity [4] scale appropriately.

Max Entropy Derivation

We will consider a set of $j \in 1, \ldots, N_{\mathrm{Prot}}$ proteins each of length N_j. To each protein an ensemble of $i \in 1, \ldots, N_{\mathrm{Seq}}$ sequences is designed. Hence the probability $P(S_i, \Gamma_j)$ of having a sequence i on structure j is given by the Boltzmann weight:

$$P(S_i, \Gamma_j) = \frac{\exp^{-\beta E(\epsilon, E_{\mathrm{HOH}}, \Omega)[S_i, \Gamma_j]}}{\sum_i^{N_{\mathrm{Seq}}} \exp^{-\beta E(\epsilon, E_{\mathrm{HOH}}, \Omega)[S_i, \Gamma_j]}}, \tag{S5}$$

where $E(\epsilon, E_{\mathrm{HOH}}, \Omega)[S_i, \Gamma_j]$ is the residue interactions energy function in Eq. S4 calculated for sequence S_i and conformation Γ_j. It is important to stress that since during the design procedure the backbone degrees of freedom of the target structure are kept frozen, the backbone hydrogen bonds are also frozen and so do not play a role in the optimization procedure.

The objective is to determine the unknown parameters ϵ, E_{HOH}, and Ω following the MEP procedure. Hence, we need to maximize the entropy associated to the probability distribution $P(S_i, \Gamma_j)$, under the constraint that the designed sequences are as close as possible to the natural ones. Our choice for the constraints is based on the results presented in our previous publication [1], were we only optimized E_{HOH}, and Ω to match the HP profiles among designed and natural sequences for just protein 1ctf. No optimization of the interaction matrix ϵ was performed. Nevertheless, the matching was successful, and the novel energy function was capable of refolding the natural sequence of 1ctf. Let us start by

introducing the entropy S associated to $P(S_i,\Gamma_j)$

$$S = -\sum_j^{N_{\mathrm{Prot}}}\sum_i^{N_{\mathrm{Seq}}} P(S_i,\Gamma_j)\ln P(S_i,\Gamma_j) \tag{S6}$$

For simplicity we show the derivation of the maximum entropy principle for $P(S_i,\Gamma_j)$ only under the constraints that designed proteins have an average HP profile close to the natural one, and that $P(S_i,\Gamma_j)$ is normalized. In order to do so we are going to make use of the Lagrange multiplier method, which means that the maximization process is equivalent to find the extremal of the function Λ defined as follows:

$$\Lambda = S + \sum_j^{N_{\mathrm{Prot}}}\sum_k^{L}\lambda_{jk}\left(\sum_i^{N_{\mathrm{Seq}}} P(S_i,\Gamma_j)\alpha_k^i - \alpha_k^{\mathrm{Real}_j}\right) + \sum_j^{N_{\mathrm{Prot}}}\gamma_j\left(Z_j - 1\right) \tag{S7}$$

where the λ_{jk} and γ_j are the Lagrange multiplier and $Z_j = \sum_i^{N_{\mathrm{Seq}}} P(S_i,\Gamma_j)$ is the partition function. We now have to derive the Λ function with respect to P keeping the Lagrange multiplier constant and look for the maximum.

$$\frac{d\Lambda}{dP(S_i,\Gamma_j)} = -\ln P(S_i,\Gamma_j) + 1 + \sum_k^{L}\lambda_{jk}\alpha_k^i + \gamma_j = 0 \tag{S8}$$

which gives for the $P(S_i,\Gamma_j)$ the following expression

$$P(S_i,\Gamma_j) = e^{\gamma_j+1}e^{\sum_k^L \lambda_{jk}\alpha_k^i} \tag{S9}$$

$$\sum_i^{N_{\mathrm{Seq}}} P(S_i,\Gamma_j) = 1 \tag{S10}$$

$$e^{\gamma_j+1} = \frac{1}{\sum_i^{N_{\mathrm{Seq}}} e^{\sum_k^L \lambda_{jk}\alpha_k^i}} \tag{S11}$$

$$\begin{aligned} P(S_i,\Gamma_j) &= \frac{e^{\sum_k^L \lambda_{jk}\alpha_k^i}}{\sum_i^{N_{\mathrm{Seq}}} e^{\sum_k^L \lambda_{jk}\alpha_k^i}} \\ &= \frac{e^{\sum_k^L \lambda_{jk}\alpha_k^i}}{Z_j'} \end{aligned} \tag{S12}$$

Now that we have the relation that connects the Lagrange multiplier with the probability distribution P we can express the function Λ in terms of the Lagrange multipliers only

$$\begin{aligned} \Lambda = &-\sum_j^{N_{\mathrm{Prot}}}\sum_i^{N_{\mathrm{Seq}}}\frac{e^{\sum_k^L \lambda_{jk}\alpha_k^i}}{Z_j'}\left(\sum_k^L \lambda_{jk}\alpha_k^i - \ln Z_j'\right) \\ &+ \sum_j^{N_{\mathrm{Prot}}}\sum_k^{L}\lambda_{jk}\left(\sum_i^{N_{\mathrm{Seq}}}\frac{e^{\sum_k^L \lambda_{jk}\alpha_k^i}}{Z_j'}\alpha_k^i - \alpha_k^{\mathrm{Real}_j}\right) \end{aligned} \tag{S13}$$

If we now rearrange the terms inside the sums

$$\begin{aligned}
\Lambda = & -\sum_{j}^{N_{\text{Prot}}}\sum_{i}^{N_{\text{Seq}}}\frac{e^{\sum_k^L \lambda_{jk}\alpha_k^i}}{Z'_j}\sum_{k}^{L}\lambda_{jk}\alpha_k^i + \sum_{j}^{N_{\text{Prot}}}\ln Z'_j \\
& + \sum_{j}^{N_{\text{Prot}}}\sum_{k}^{L}\lambda_{jk}\sum_{i}^{N_{\text{Seq}}}\frac{e^{\sum_k^L \lambda_{jk}\alpha_k^i}}{Z'_j}\alpha_k^i - \sum_{j}^{N_{\text{Prot}}}\sum_{k}^{L}\lambda_{jk}\alpha_k^{\text{Real}_j} \\
= & -\sum_{j}^{N_{\text{Prot}}}\sum_{k}^{L}\lambda_{jk}\sum_{i}^{N_{\text{Seq}}}\frac{e^{\sum_k^L \lambda_{jk}\alpha_k^i}}{Z'_j}\alpha_k^i + \sum_{j}^{N_{\text{Prot}}}\ln Z'_j \\
& + \sum_{j}^{N_{\text{Prot}}}\sum_{k}^{L}\lambda_{jk}\sum_{i}^{N_{\text{Seq}}}\frac{e^{\sum_k^L \lambda_{jk}\alpha_k^i}}{Z'_j}\alpha_k^i - \sum_{j}^{N_{\text{Prot}}}\sum_{k}^{L}\lambda_{jk}\alpha_k^{\text{Real}_j} \\
= & \sum_{j}^{N_{\text{Prot}}}\ln Z'_j - \sum_{j}^{N_{\text{Prot}}}\sum_{k}^{L}\lambda_{jk}\alpha_k^{\text{Real}_j}
\end{aligned} \tag{S14}$$

We can now derive the condition for the Lagrange multiplier to maximize the functional Λ

$$\begin{aligned}
\frac{\partial\Lambda}{\partial\lambda_{jk}} & = \frac{1}{Z'_j}\frac{\partial Z'_j}{\partial\lambda_{jk}} - \alpha_k^{\text{Real}_j} \\
& = \frac{1}{Z'_j}\sum_{i}^{N_{\text{Seq}}}\alpha_k^i e^{\sum_k^L \lambda_{jk}\alpha_k^i} - \alpha_k^{\text{Real}_j} = 0
\end{aligned} \tag{S15}$$

This result can be interpreted as follows: the distribution generated by the Lagrange multiplier that makes the average hydrophobic profile equal to the natural one, is also the one that maximizes the entropy. Hence, we selected the following scoring function:

$$\begin{aligned}
F_{\text{score}} = & \sum_{j}^{N_{\text{Prot}}}\sum_{k}^{N_j}\left(\sum_{i}^{N_{\text{Seq}}}P(S_i,\Gamma_j)E_{\text{Sol}}^{ik} - E_{\text{Sol}}^{\text{Real}_{jk}}\right)^2 + \\
& \sum_{j}^{N_{\text{Prot}}}\sum_{k}^{N_j}\left(\sum_{i}^{N_{\text{Seq}}}P(S_i,\Gamma_j)\gamma_k^i - \gamma_k^{\text{Real}_j}\right)^2 + \\
& E_{\text{Shannon}}\sum H(\epsilon)\log H(\epsilon)
\end{aligned} \tag{S16}$$

where E_{Sol} is the hydrophobicity scale per residue (see Eq. S3), $\gamma_k^i = \sum_k^{N_j}\epsilon_{kl}\Gamma(r_{kl}) + E_{\text{HOH}}\sum_k^N E_{\text{Sol}}$'s are the contribution to the total energy per residue, and $E_{\text{Shannon}} = 8.0$. Hence, the scoring function is just a comparison of the designed hydrophobic and energy profiles averaged over all designed sequences to the real profile. Since protein design can be

performed in parallel, each averaging step is very fast. Nevertheless, before reaching total convergence of the parameters we had to perform $\sim 10^8$ iterations for a total of three weeks of computations on 250 AMD Opteron 6132 HE, 2.2 GHz each with 8 cores . It is important to note that, in order to speed up the sampling of such systems, inevitably rich in local energy traps, we used the Virtual Move Parallel Tempering scheme [5]. It should be noted that the in the MEP the exact solution for the Lagrange multipliers should be found with a convex minimization procedure. We choose instead to approximately satisfy the MEP with an MC scheme in order to sample the fluctuations around the optimal solution.

Free Energy Calculations

In order to test the folding behaviour of the designed and natural sequences we used two algorithms that we refer to as DESIGN and FOLDING to design new sequences and to compute the refolding free energies of both designed and natural sequences. Both DESIGN and FOLDING are described in our previous work [1]. The FOLDING simulation starts from a fully stretched chain and the configurational space is explored by means of pivot moves around the dihedral angles and by a crankshaft move [6]. The latter consists in a rotation of all the atoms between two randomly chosen C_α carbons. The rotation is then performed around the axis connecting the two C_α atoms. When this move is performed on two consecutive C_α's it rotates the rigid body composed by the atoms C,N, H and O, and helps to equilibrate locally the hydrogen bonds. It is important to notice that the crankshaft move will distort the $\widehat{CC_\alpha N}$ angle which is kept close to his equilibrium value by a strong (50 $k_B T/\text{Å}^2$ elastic constant) spring, hence moves that case large distortion of the angle are rejected. During a FOLDING simulation the sampled configurations are grouped in ensembles defined by the free energy $F\,(\text{DRMSD})$ as a function of DRMSD to the target structure. For a well-designed sequence and a natural folding sequence will fold into structures fluctuating around the target structure corresponding to DRMSD = 0. In order to increase the sampling of the free energy for the FOLDING simulations we will use the Virtual Move Parallel Tempering (VMPT) scheme [5] for the range of temperatures $[0.40, 0.31, 0.22, 0.20, 0.18, 0.16, 0.13, 0.12, 0.11, 0.10, 0.09, 0.08, 0.07, 0.06, 0.05, 0.04]$. We could not determine precisely the folding temperatures T_F for all the proteins studied and we only estimated it to be between the highest temperature where the protein is still

folded and the one above where the protein is unfolded. However and exact estimate is not essential for the scope of this work, but we plan in the future to study the folding transition more in detail. What we observed in our previous publication [1] for the protein 1CTF is that close to T_F folded state has approximately the same free energy as the high temperature disordered globular state S1. It is important to stress that the Virtual Move Parallel Tempering biasing scheme is designed to allow the system to explore as much as possible of the free energy landscape and not to drive the system towards the minimum, which means that even if we would start form the folded configuration, the simulation would quickly evolve away from the global minimum.

DRMSD

The distance root mean square displacement is a standard collective variable used in the field of protein folding to measure the state of the folding transition. When a target structure is given the DRMSD is defined as

$$\text{DRMSD} = \frac{1}{N}\sqrt{\sum_{ij}\left(|\Delta\vec{r}_{ij}| - |\Delta\vec{r}_{ij}^{\,T}|\right)^2}, \tag{S17}$$

where $\vec{r}_{ij}$ is the distance between the sphere i and j while $\vec{r}_{ij}^{\,T}$ is the same distance calculated over the target structure, and N is the chain length. According to Eq.(S17), DRMSD$=0$ is possible only when the chain and the target structures are identical. Any structural difference will correspond to larger values of DRMSD, and the larger the value of DRMSD the larger is the number of structures that share the same DRMSD from the target. In order to justify the use of DRMSD instead of more commonly use of RMSD we measured the correlation between the two collective variables. In Fig S2(b) we compare the two quantities and we measured the correlation faction near the free energy minimum. The results indicate that the two quantities are highly correlated for DRMSD$> 1.5\mathring{A}$ which is compatible with the current resolution of the caterpillar model.

---

* ivan.coluzza@univie.ac.at

[1] I. Coluzza, PloS one **6**, e20853 (2011).
[2] T. E. Creighton, *Proteins: structures and molecular properties* (W. H. Freeman, 1993), ISBN 071677030X.
[3] A. Irbäck, F. Sjunnesson, and S. Wallin, Proceedings of the National Academy of Sciences of the United States of America **97**, 13614 (2000).

[4] R. F. Dolittle, *In Predictions of Protein Structure and the Principles of Protein Conformation* (Springer, 1989).

[5] I. Coluzza and D. Frenkel, Chemphyschem : a European journal of chemical physics and physical chemistry **6**, 1779 (2005).

[6] D. Frenkel and B. Smit, *Understand molecular simulations* (2002).

[7] Y. Zhang and J. Skolnick, Proteins **57**, 702 (2004).

[8] J. Xu and Y. Zhang, Bioinformatics (Oxford, England) **26**, 889 (2010).
